# Changes in Sensitivity of Reward and Motor Behavior to Dopaminergic, Glutamatergic, and Cholinergic Drugs in a Mouse Model of Fragile X Syndrome

**DOI:** 10.1371/journal.pone.0077896

**Published:** 2013-10-18

**Authors:** Eric W. Fish, Michael C. Krouse, Sierra J. Stringfield, Jeffrey F. DiBerto, J. Elliott Robinson, C. J. Malanga

**Affiliations:** 1 Department of Neurology, University of North Carolina at Chapel Hill, Chapel Hill, North Carolina, United States of America; 2 Curriculum in Neurobiology, University of North Carolina at Chapel Hill, Chapel Hill, North Carolina, United States of America; 3 Carolina Institute for Developmental Disabilities, University of North Carolina at Chapel Hill, Chapel Hill, North Carolina, United States of America; University of Colorado, United States of America

## Abstract

Fragile X syndrome (FXS) is a leading cause of intellectual disability. FXS is caused by loss of function of the FMR1 gene, and mice in which *Fmr1* has been inactivated have been used extensively as a preclinical model for FXS. We investigated the behavioral pharmacology of drugs acting through dopaminergic, glutamatergic, and cholinergic systems in fragile X (*Fmr1* ^-/Y^) mice with intracranial self-stimulation (ICSS) and locomotor activity measurements. We also measured brain expression of tyrosine hydroxylase (TH), the rate-limiting enzyme in dopamine biosynthesis. *Fmr1* ^-/Y^ mice were more sensitive than wild type mice to the rewarding effects of cocaine, but less sensitive to its locomotor stimulating effects. Anhedonic but not motor depressant effects of the atypical neuroleptic, aripiprazole, were reduced in *Fmr1* ^-/Y^ mice. The mGluR5-selective antagonist, 6-methyl-2-(phenylethynyl)pyridine (MPEP), was more rewarding and the preferential M1 antagonist, trihexyphenidyl, was less rewarding in *Fmr1* ^-/Y^ than wild type mice. Motor stimulation by MPEP was unchanged, but stimulation by trihexyphenidyl was markedly increased, in *Fmr1* ^-/Y^ mice. Numbers of midbrain TH+ neurons in the ventral tegmental area were unchanged, but were lower in the substantia nigra of *Fmr1* ^-/Y^ mice, although no changes in TH levels were found in their forebrain targets. The data are discussed in the context of known changes in the synaptic physiology and pharmacology of limbic motor systems in the *Fmr1* ^-/Y^ mouse model. Preclinical findings suggest that drugs acting through multiple neurotransmitter systems may be necessary to fully address abnormal behaviors in individuals with FXS.

## Introduction

Fragile X syndrome (FXS) is the most common inherited cause of intellectual disability and the most common single-gene defect identified in autism [1]. Approximately one-third of patients with FXS are eventually diagnosed with autism spectrum disorder [2], and it is estimated that up to 6-8% of children diagnosed with autism carry mutations in the X-linked FMR1 gene [3]. Patients with FXS often have deficits in verbal and performance skills, spatial reasoning, and short term memory, as well as attention deficits and hyperactivity, stereotypic movements, and atypical social development [4-7]. FXS results from inappropriate transcriptional silencing of the FMR1 gene [8] and failure to express its product, FMRP (fragile X mental retardation protein), an RNA-binding protein that represses local protein synthesis [9]. Mice lacking the *Fmr1* gene model aspects of the pathophysiology and many of the abnormal behaviors seen in FXS and autism, including cognitive impairments [10-13], increased spontaneous motor activity [14-16] (but see 17,18), increased seizure susceptibility [19,20]; and altered social behaviors [17,21-23].

FMRP opposes signaling through G protein-coupled receptors (GPCRs) acting through the Gqα-subunit, including group I metabotropic glutamate (mGluR1/5) and M1 muscarinic acetylcholine (mAChR1) receptors. Gq-coupled GPCRs signal through phospholipase-C (PLC) and phosphoinositide 3-kinase (PI3K), influencing local protein synthesis through both the Akt/mTOR and MEK/ERK pathways (reviewed in 24). In dendritic spines, activity at these receptors in response to stimuli facilitates local synaptic protein translation; and lack of FMRP therefore leads to abnormally exaggerated experience and protein synthesis-dependent synaptic plasticity [25-28]. Spine development is impaired such that spines are longer and thinner, retaining a more immature form, and do not undergo normal experience-dependent modification of size, shape, or number [29,30]. PLC signaling is important in activity-dependent spine development, supporting findings that mGluR antagonists normalize spine morphology in *fmr1*-null mice [31,32]. Heterodimeric D1/D2 dopamine receptors also activate PLC through Gq [33,34], but whether this signaling mechanism is affected in *Fmr1*-null mice is not yet known.

Although the pathophysiological mechanisms in FXS are some of the most understood among the genetic synaptopathies, therapy for this disorder currently consists of symptom management and not pharmacological correction or reversal of synaptic changes due to loss of FMRP. Both mAChR and mGluR-dependent LTD is enhanced in hippocampal neurons [28]. Antagonism of mGluRs has been proposed as a rational therapy for FXS [35], and preclinical studies have shown that mGluR5 antagonists can partially correct some abnormal behaviors in *Fmr1*-null mice, including increased open-field exploration, impaired rotarod performance, and decreased prepulse inhibition, although results have been mixed [18,36,37]. While still under clinical development, no mGluR antagonists are yet approved for human use. The M1 muscarinic receptor antagonist, dicyclomine, and the M4 antagonist, tropicamide, have also been shown to partially improve abnormal behaviors and seizure susceptibility in *Fmr1*-null mice [38,39].

The dopamine, glutamate, and acetylcholine systems in the brain are all affected in mice lacking *Fmr1*. Dopamine in particular is important for the initiation and reinforcement of motivated behaviors. Mice lacking *Fmr1* have increased dopamine turnover [40] but decreased amphetamine-stimulated dopamine release in the dorsal striatum [15], which correlates with decreased sensitivity to amphetamine-induced motor stereotypies [41]; as well as increased dopamine release in the prefrontal cortex [15]. The postsynaptic effects of dopamine D1 receptor activity on AMPA-type glutamate receptor function are also reduced in both prefrontal cortex and striatum [14,42].

There are relatively fewer behavioral or neurochemical studies on limbic motor system function in *Fmr1*-null mice than in hippocampus or neocortex. Given the critical involvement of limbic circuitry in motivation and reinforcement, changes in social integrative behavior and motor learning in FXS may be affected by underlying deficits in limbic brain reward circuitry as well as in cortex involved in memory and higher cognitive functions. Although two studies have shown normal acquisition of operant behavior using sucrose or food reinforcement in *Fmr1*-null mice [10,43], the neural mechanisms underlying motivation and reward have not been explored in depth in this model. Imaging studies have identified alterations in both morphology [44-46] and activation patterns [47,48] in the striatum of FXS patients, but the function of dopaminergic projections from the midbrain substantia nigra pars compacta (SNc) and ventral tegmental area (VTA) to their forebrain targets in the dorsal striatum and nucleus accumbens (NAc) have been less extensively investigated than cortical circuits in *Fmr1*-null mice. Drugs that directly affect the dopamine system, including atypical neuroleptics such as aripiprazole, are of interest for the management of affective and behavioral symptoms in FXS [49,50]. Cholinergic mechanisms in mesolimbic and nigrostriatal motor function, in which interactions with the dopamine system shape striatal output [51-54], are largely unexplored in this model.

The goal of the current study was to characterize limbic motor circuitry with behavioral and neurochemical methods in *Fmr1*-null mice. Intracranial self-stimulation (ICSS) is an operant behavior in which animals perform a task for reinforcement by electrical brain stimulation reward (BSR). The predictable effects on BSR of drugs acting through dopamine, glutamate, or acetylcholine receptors can be compared between genotypes, and we have previously used this approach to investigate pharmacological mechanisms in other monogenic neurodevelopmental disorders [55]. We hypothesize that *Fmr1*-null mice will show increased sensitivity to drugs that enhance the rewarding value of BSR and, conversely, decreased sensitivity to the reward-devaluing effects of drugs that diminish BSR. Experiments measuring the effects of the atypical neuroleptic aripiprazole, the mGluR5 antagonist MPEP, and the preferential M1 antagonist trihexyphenidyl on locomotor behavior were also performed to further differentiate drug effects on global motor function from effects specific to operant behavior. To determine if absence of *Fmr1* alters dopaminergic neurons originating in the SNc and VTA, tyrosine hydroxylase immunoreactivity was also quantified by design-based stereology in midbrain histological sections and by western blot in tissue homogenates from dorsal striatum and NAc.

## Methods

### Ethics Statement

All procedures were approved by The Institutional Animal Care and Use Committee (IACUC) of the University of North Carolina at Chapel Hill (Protocol 12-146.0) and were conducted according to the Guide for the Care and Use of Laboratory Animals (NIH publication No. 85-23, revised 2011).

### Mice

Male wild type (WT) and *Fmr1*-null mice (*Fmr1* ^-/Y^) were bred on site from founders generously provided by Dr. William Greenough at the Beckman Institute, University of Illinois (see 56,57 for information on mouse origins). Mice were housed in polycarbonate cages (28 × 17 × 14 cm) with wire lids and cob bedding that was changed weekly. Mice used for ICSS (*n* = 31 WT, 29 *Fmr1* ^-/Y^) and locomotor activity (*n* = 11 WT, 11 *Fmr1* ^-/Y^) experiments were housed individually while the mice for tyrosine hydroxylase (TH) immunohistochemistry (*n* = 5 WT, 7 *Fmr1* ^-/Y^) and protein determinations (*n* = 6 WT, 9 *Fmr1* ^-/Y^) were housed in groups of two to four. All mice had free access to food and water and were allowed to acclimate to the vivarium for at least one week prior to experiments. The vivarium was at 21°C with a 12-hour light cycle (lights on at 8:00 PM); all procedures were conducted in the dark phase between 8:30 AM and 4:30 PM. All behavioral experiments were performed with the experimenter blinded to mouse genotype.

### Drugs

Cocaine hydrochloride (Sigma-Aldrich, St. Louis MO) and the mGluR5-selective antagonist, MPEP (6-methyl-2-(phenylethynyl)pyridine hydrochloride, Tocris, Bristol UK), were dissolved in saline, and doses were calculated as the free base. The partially selective mAChR1 (M1) antagonist, trihexyphenidyl (1-cyclohexyl-1-phenyl-3-(1-piperidyl)propan-1-ol, Sigma-Aldrich), was dissolved in dH_2_O. The atypical neuroleptic, aripiprazole (7-{4-[4-(2,3-dichlorophenyl)piperazin-1-yl]butoxy}-3,4-dihydroquinolin-2(1H)-one, generously provided by Bristol Myers Squibb/Otsuka Pharmaceuticals), was dissolved in glacial acetic acid and then diluted in a 2% Tween-20 solution to a final pH of 4.3. All drugs were administered intraperitoneally through a 27-gauge needle in a volume of 1ml/100g body weight.

### Electrode Implantation

Intracranial self-stimulation (ICSS) experiments were conducted in adult (P55+) male mice that were anesthetized (120 mg/kg ketamine and 18 mg/kg xylazine; Sigma-Aldrich) and stereotaxically-implanted in the right medial forebrain bundle at the level of the lateral hypothalamus (coordinates: AP -1.3, ML -1.0, DV -5.2; [58]) with insulated monopolar stainless steel electrodes (0.28 mm diameter; Plastics One, Roanoke VA). The electrode was grounded to the skull with a stainless steel screw and secured to the skull with dental cement. Following surgery, animals were returned to their cages for one week of recovery. ICSS electrode placements are shown in the **Supporting Information,** Figure **S1**.

### Intracranial Self-Stimulation

ICSS experiments were performed as previously described [55] in sound-attenuating chambers (16 × 14 × 13 in; MedAssociates, St Albans VT) containing an operant conditioning chamber with a grid floor (ENV-005A; MedAssociates), wheel manipulandum (ENV-113AM; MedAssociates), and house light (ENV-315W; MedAssociates). Electrical stimulation was controlled by MED-PC software for Windows (v4.1; MedAssociates) and a stimulator (PHM-150B/2; MedAssociates) connected to a swivel commutator and insulated wire (Plastics One) that attached to the stimulating electrode. A computer interface was connected to each box to record responses (1 response = ¼ turn of the wheel manipulandum), activate the house light, and issue electrical current (brain stimulation reward, BSR). 

Following recovery after surgery, mice were conditioned to respond for BSR. Each stimulation consisted of a 500 ms train of unipolar cathodal square-wave current pulses delivered at a trial-dependent frequency with a 100 μs pulse width. During the 500 ms stimulation period, wheel responses were recorded but did not earn additional stimulation. Each response was accompanied by illumination of the house light for 500 ms as a secondary reinforcer. Initially, optimal stimulus intensity to sustain responding >40 responses/min at 158 Hz was determined for each mouse (-60 to -190 μA) and was kept constant for each mouse for all experiments.

Mice were then trained with a series of stimulus frequencies in descending order from 158 Hz in 0.05 log_10_ increments (i.e., log_10_[112 Hz] = 2.05; log_10_[100 Hz] = 2.00, etc.). Each frequency trial began with a 5 sec time-out, during which responses earned no stimulation, followed by five non-contingent priming stimulations (1 train/sec) and access to BSR at that stimulus frequency for 50 sec, during which responses were measured. All mice were trained to complete four series of 15 trial frequencies (i.e., 1 hour) daily. The frequency range was adjusted for each mouse such that only the highest 4-5 frequencies sustained responding, and was kept constant for each mouse for all experiments. Maximum stimulus frequencies ranged from 158 Hz to 100 Hz and did not differ between genotypes (135 ± 4.1 Hz for WT vs. 139 ± 3.8 Hz for *Fmr1* ^-/Y^ mice, *p* = 0.49). Training continued until daily BSR threshold (θ_0_, [59]) calculated as the average of the second, third, and fourth daily series, varied by less than ± 10% over three consecutive days. 

During each testing session, mice first responded in three consecutive series of 15 descending frequency trials. Because responding during the first series of each test day is variable, daily baseline BSR thresholds (θ_0_, [59]) were averaged from responses during the second and third series. After baseline determinations, mice were removed from the conditioning chambers, injected intraperitoneally with saline or drug vehicle (for aripiprazole and trihexyphenidyl experiments), cocaine (1.0, 3.0, or 10.0 mg/kg, i.p.), aripiprazole (0.03, 0.1, or 0.3 mg/kg, i.p.), MPEP (3.0, 5.6, or 10.0 mg/kg, i.p.), or trihexyphenidyl (3.0, 10.0, or 30.0 mg/kg, i.p.), and returned to the conditioning chambers for 1 hour (i.e., four 15-minute response series). The order of each drug dose was counter-balanced across mice and each drug dose was separated by a vehicle injection. For experiments with aripiprazole only, because pilot studies had indicated a longer onset of action than for the other drugs tested, mice were injected and placed in their home cage for 15 minutes before being returned to the operant conditioning chamber. BSR threshold and maximum operant response rate (MAX) were calculated for each post-injection series and expressed as a percentage of the daily pre-injection baseline. 

### Locomotor Activity

Adult male mice (P89-193, mean = P139) were placed in the centers of 28 x 28 cm Plexiglas chambers (ENV-1510; Med Associates) containing two sets of 16 pulse-modulated infrared photobeams. Each photobeam interruption was relayed to a computer running MedAssociates IV software that determined the position of the mouse every 100 ms and calculated the total distance traveled (cm). Locomotor behavior experiments were conducted three days a week and separated by at least 48 hours. On each test session, the mice were placed into the activity chambers for 45 min then removed, injected, and returned to the chambers for 60 min in order to directly compare drug effects on locomotor behavior and ICSS. As in ICSS experiments, for experiments with aripiprazole and its vehicle only, mice were injected and placed in their home cages for 15 min before being returned to the activity chambers. The first three experimental days habituated the mice to the apparatus and to handling required for injection. Saline injections were given on the next three experimental days to habituate the mice to intraperitoneal injection. For drug testing, each drug dose was separated by at least one vehicle injection. On the basis of results from ICSS experiments, all mice received the following drug treatments in the following order: cocaine (3.0 mg/kg, i.p.); aripiprazole (0.1 mg/kg, i.p.); MPEP (5.6 mg/kg, i.p.); and trihexyphenidyl (10.0 mg/kg, i.p.), cocaine (1.0 mg/kg), and cocaine (10.0 mg/kg). 

### Tyrosine Hydroxylase Immunohistochemistry

Experimentally naïve adult male mice (P120-126) were deeply anesthetized (pentobarbital 120 mg/kg, i.p.) and perfused transcardially with 0.1 M phosphate-buffered saline (PBS, pH 7.4), followed by 4% paraformaldehyde in 0.1 M PBS. All brains were post-fixed by submersion in the same fixative for 24 hours, then cryoprotected in 10% sucrose for 24 hours, followed by 30% sucrose for 24 hours. Brains were sectioned (50 μm, coronal) on a sliding microtome and stored in cryoprotectant (1.0% w/v polyvinylpyrrolidone, 30% w/v sucrose, and 30% v/v ethylene glycol in 0.1 M PBS). Endogenous peroxidase activity was quenched with 1% hydrogen peroxide in 0.1 M PBS for 30 minutes at room temperature. Sections were blocked in 5% normal goat serum (NGS) and 2% Triton X-100 in 0.1 M PBS for 24 hours at room temperature, then incubated in rabbit-α-rat tyrosine hydroxylase (TH) primary Ab (1:500; AB152; Millipore, Billerica MA) in 2% NGS and 2% Triton for 4 days at 4°C. Sections were washed (x3) in 0.1 M PBS and incubated with HRP-conjugated goat-α-rabbit secondary Ab (1:250; 31460; Thermo Scientific, Rockford IL) in 2% NGS and 2% Triton at room temperature for 3 hours. Reaction product was visualized with a DAB peroxidase substrate kit (SK-4100; Vector Laboratories, Burlingame CA). Sections were mounted on potassium dichromate/gelatin-subbed slides and counterstained with cresyl violet for Nissl.

### Stereological Estimation of Cell Numbers

The total number of TH-positive cells in the substantia nigra pars compacta (SNc) and ventral tegmental area (VTA) were estimated with design-based stereology using StereoInvestigator v9.14 (MBF Bioscience, Williston VT) on a Microphot FXA microscope (Nikon, Japan). Every fourth section (section interval = 4) was immunostained and counted, yielding on average eight midbrain sections per animal. The borders of the SNc and the VTA at all rostrocaudal levels in the midbrain were delineated at low (4x) magnification based on the standard mouse atlas [58]. For purposes of this study, the VTA and SNc were divided by a line connecting the medial border of the medial lemniscus, dorsally, to the medial edge of the corticospinal tract, ventrally, in all sections. Counting frames (50 µm x 50 µm) were randomly placed and systematically moved through a sampling grid (120 µm x 120 µm) by the software via a motorized stage (Ludl Electronic Products, Hawthorne NY). An optical disector height of 15.0 µm flanked by 2.0 µm guard zones on top and bottom was applied to all counting frames based on average section shrinkage estimates. Brightfield counting under direct visualization was performed at 100x oil magnification (NA = 1.4). Estimates of the total numbers of TH-immunostained neurons were calculated using the optical fractionator method [60]. For all samples, coefficients of error ≤ 0.10 (Gundersen, m = 1) were accepted [61].

### Tyrosine Hydroxylase Western Blots

Experimentally naïve adult male mice (P81-166, mean = P114) were deeply anesthetized with isoflurane inhalation and decapitated. Brains were collected, flash-frozen in isopentane at -40°C, and stored at -80°C. Coronal tissue slabs (1.0 mm) were collected from frozen brains using a cryostat at -15°C (Leica, Buffalo Grove IL). Frozen tissue punches (1.0 mm dia.) were taken from slabs containing the NAc and dorsal striatum and homogenized by ultrasonification (Branson Ultrasonics, Danbury CT) in a buffer containing 5% (w/v) sodium dodecyl sulfate in 0.05M Tris buffer (pH = 7.4), a Complete Protease Inhibitor Cocktail tablet (Roche, Florence SC) and 500 μL each of phosphatase inhibitor 2 and 3 (Sigma-Aldrich). Protein concentration was measured using a calorimetric assay kit (Pierce BCA; Thermo Scientific). Protein (5 μg) was diluted 4:1 with lithium dodecyl sulfate sample buffer (40–70% glycerol) and 10:1 with NuPAGE (Invitrogen, Carlsbad CA) and heated at 70°C for 5 minutes. Samples were injected onto a Nu-Page 4-15% Tris-Glycine polyacrylamide gel (BioRad, Hercules CA) for gel electrophoresis separation, and transferred to a nitrocellulose membrane using an iBlot dry blotting system (Invitrogen). Membranes were blocked with 3% bovine serum albumin (Sigma-Aldrich) before incubation with rabbit-α-rat TH primary Ab (1:2000; AB152, Millipore) overnight at 4°C. Membranes were washed before incubation with an HRP-conjugated goat-α-rabbit secondary Ab (1:10,000; Jackson ImmunoResearch, West Grove PA) for 1 hour at room temperature. Membranes were then incubated for 5 minutes in Amersham ECL Prime Western Blotting Detection Reagent (GE, Piscataway NJ). Bands were visualized on an ImageQuant LAS4000 system (GE) and quantified using optical density measurements (ImageQuantTL v7.0, GE). A similar method was used to detect β-actin using a mouse monoclonal anti-β-actin primary Ab (1:5000; Millipore) for 1 hour at room temperature and an HRP-conjugated goat-α-mouse secondary Ab (1:10,000; Jackson ImmunoResearch) for 1 hour at room temperature. Data were expressed as ratios of TH:β-actin for each sample.

### Data Analysis

For ICSS experiments, statistical comparisons of baseline BSR thresholds and maximum operant response rates (MAX) were performed using Student’s t-tests for unmatched samples (two-tailed). Comparisons of drug effects on BSR threshold and MAX in WT and *Fmr1* ^-/Y^ mice were performed by two-way repeated measures ANOVAs (dose x genotype) for each 15-minute post-injection series followed by *post hoc* Bonferroni corrected comparisons to control (vehicle or WT). When main effects of genotype but no dose x genotype interaction was observed, one-way ANOVA for effect of dose was performed independently for each genotype to determine differences from vehicle injection. For locomotor behavior experiments, comparisons of total distance traveled and change from baseline activity were performed using two-way repeated measures ANOVA (time x genotype) at each dose with Bonferroni corrected *post hoc* comparisons to control. On the habituation days, comparison of total distance traveled before and after handling was performed with Student’s t-tests for unmatched samples (two-tailed). In the case of cocaine, two-way repeated measures ANOVA (dose x genotype) with pairwise Bonferroni corrections *post hoc* were also performed. For stereological estimates of TH+ SNc and VTA neurons and for TH:β-Actin ratios in tissue homogenates of dorsal striatum and NAc, Levene’s *F*-tests for equality of variance were followed with Student’s *t*-tests for unmatched samples (two-tailed). For all statistical comparisons, *p* < 0.05 was considered significant. 

## Results

### Baseline ICSS and Locomotor Behavior

**Figure 1:**
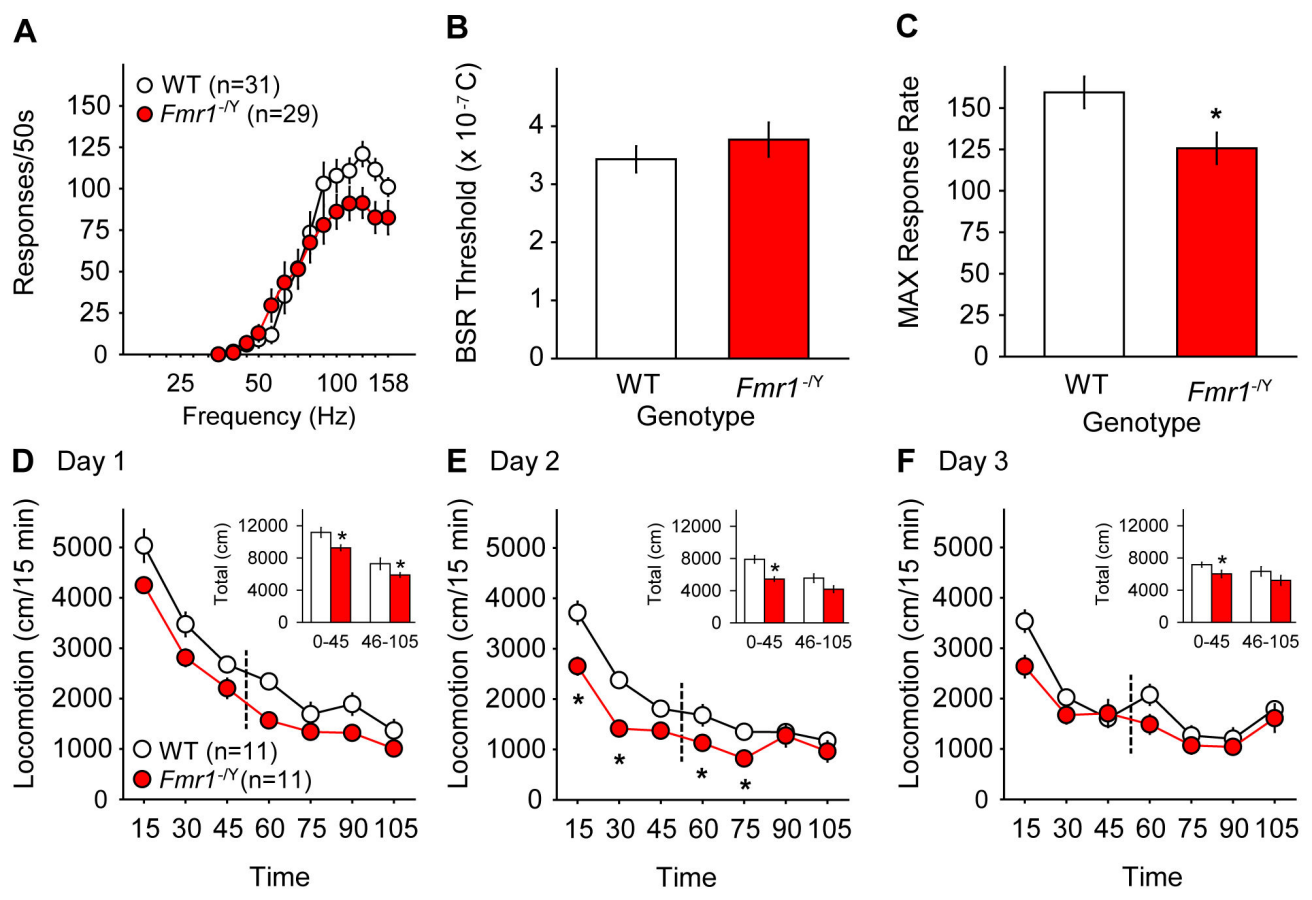
Comparison of ICSS and locomotor behavior in wild type (WT, *white* *circles*) and *Fmr1* ^-/Y^ mice (red circles). **A**. Both *Fmr1* ^-/Y^ and WT mice responded for BSR in a frequency-dependent manner. Values are mean number of responses per 50 sec access to BSR at each stimulus frequency ± SEM. **B**. BSR sensitivity expressed as electrical charge delivery at baseline BSR threshold frequency (θ_0_) did not differ between WT (white bars) and *Fmr1* ^-/Y^ mice (red bars). Values are mean charge in Coulombs ± SEM. **C**. Baseline maximum operant response rates were lower in *Fmr1* ^-/Y^ mice than WT mice. Values are mean maximum number of responses ± SEM. * = *p* < 0.05 vs. WT. **D**-**F**. Habituation to the novel locomotor apparatus and handling in WT and *Fmr1* ^-/Y^ mice. On Day 1 (D) *Fmr1* ^-/Y^ mice were less active at all time points before (15-45 min) and after handling (60-105 min), and had lower cumulative total locomotion before and after handling (inset). On Day 2 (E) *Fmr1* ^-/Y^ mice were less active prior to (inset) and for 30 min following handling. By Day 3 (F), total locomotion remained lower in *Fmr1* ^-/Y^ mice prior to handling but no difference in locomotion was seen after handling (inset). Dashed lines indicate handling time points. Values are mean distance traveled ± SEM. Asterisks (*) indicate *p* < 0.05 vs. WT.

Throughout all intracranial self-stimulation (ICSS) experiments, mice showed frequency-dependent responding for brain stimulation reward (BSR; Figure **1A**). There was no difference in sensitivity to BSR itself between genotypes, as charge delivery at baseline BSR threshold frequency (θ_0_) was similar in *Fmr1* ^-/Y^ (3.8 ± 0.37 x10^-7^ C) and WT mice (3.4 ± 0.23 x10^-7^ C, Figure **1B**). However, maximum operant response rates (MAX) were lower in *Fmr1* ^-/Y^ mice (*t*_*58*_ = 2.5; *p* = 0.015; Figure **1C**). Pre-injection baseline BSR thresholds remained stable over the course of all ICSS experiments in both WT and *Fmr1* ^-/Y^ mice (**Supporting Information**, Figure **S2**).

In open-field locomotion, *Fmr1* ^-/Y^ mice were less active than WT mice during habituation to handling and the novel apparatus on the first two days of testing. On Day 1, there was a significant main effect of genotype (*F*_*1,153*_ = 10.7; *p* = 0.004; Figure **1D**), with *Fmr1* ^-/Y^ less active than WT mice across all 15-minute pre- and post-handling test periods (*p* < 0.05), as well as over the full 45 min before (*t*_*20*_ = 2.674; *p* = 0.015) and 60 min after handling (*t*_*20*_ = 2.676; *p* = 0.015; Figure **1D**, *inset*). On Day 2, there was a significant interaction between time and genotype (*F*_*6,153*_ = 4.1; *p* < 0.001; Figure **1E**), with less activity for the first 75 minutes of testing and significantly lower total activity in the 45-minute pre-handling habituation phase in *Fmr1* ^-/Y^ mice (*t*_*20*_ = 4.9; *p* < 0.001; Figure **1E**, *inset*). By Day 3 of habituation, there was less total locomotion in the 45-minute pre-handling phase, but no differences in activity after handling (Figure **1F**).

### Pharmacological Sensitivity of BSR and Locomotor Activity

#### Cocaine

**Figure 2:**
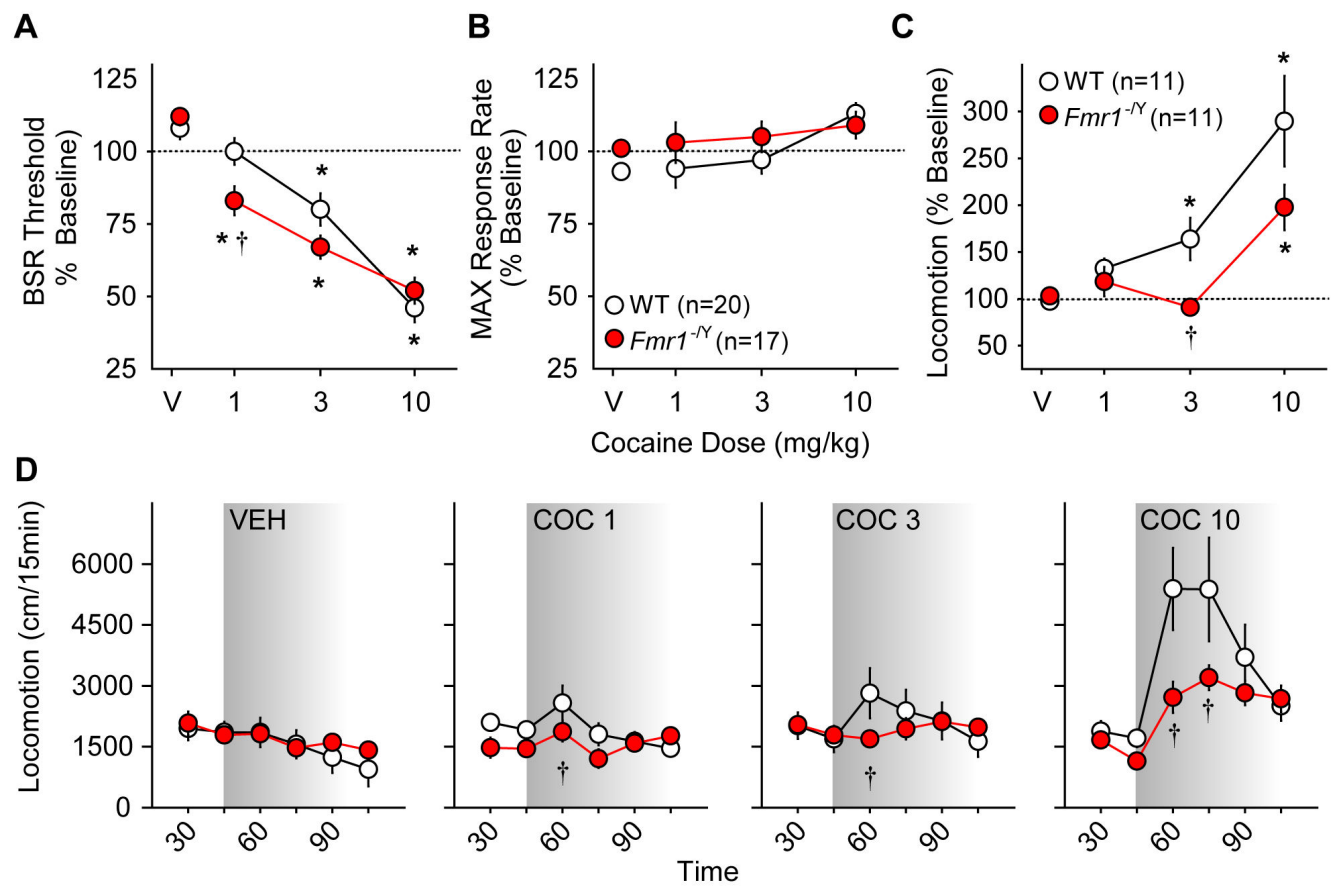
Effects of cocaine on ICSS and locomotor behavior in wild type (WT, *white* *circles*) and *Fmr1* ^-/Y^ mice (red circles). Changes in BSR threshold (**A**) and maximum operant response rate (MAX, **B**) in the first 15 minute series after i.p. cocaine injections are shown. Values are expressed as mean percentages of pre-injection baselines ± SEM. **C**. Mean change in locomotion (± SEM) as a percentage of pre-injection baseline activity after injection of saline (V) or cocaine (1.0, 3.0, or 10.0 mg/kg i.p.) in the first 15 minutes after injection. For A-C, asterisks (*) indicate *p* < 0.05 vs. vehicle (V); daggers (†) indicate *p* < 0.05 vs. WT (dose x genotype interaction *post* *hoc*). **D**. Mean distance traveled (± SEM) before and after injection of saline (VEH) or cocaine (COC, 1.0, 3.0, or 10.0 mg/kg i.p.) in 15-minute intervals. Shading indicates post-injection time points. Daggers (†) indicate *p* < 0.05 vs. WT (time x genotype interaction *post* *hoc*).

Cocaine increases the rewarding value of BSR, measured as lowering of BSR threshold. Previous studies in our laboratory have consistently shown that the maximal effect of cocaine on ICSS occurs in the first 15 minutes after injection in C57BL6/J mice [55,62,63]. There was a significant interaction between cocaine dose and genotype on changes in BSR threshold (*F*_*3,147*_ = 3.29; *p* = 0.02) in the first 15 minutes after injection (Figure **2A**). BSR thresholds were significantly lower in *Fmr1* ^-/Y^ than WT mice after 1.0 mg/kg cocaine (*p* < 0.05) and approached significance after 3.0 mg/kg cocaine (*p* = 0.053). Cocaine only increased maximum operant response rates for BSR (MAX) in the first 15 minutes in WT mice only after the 10 mg/kg dose (Figure **2B**). Significant effects of cocaine dose on BSR threshold were found in all four 15-minute testing periods after cocaine injection and on MAX in WT but not *Fmr1* ^-/Y^ mice at time points after 16-30 minutes following injection (Figures **S3A** and **S4A** and Tables **S1** and **S2**). 

Total locomotor activity after saline injection did not differ between *Fmr1* ^-/Y^ and WT mice, but significant interactions between time and genotype were found following 1.0 (*F*_*5,131*_ = 3.17; *p* = 0.01), 3.0 (*F*_*5,131*_ = 4.37; *p* = 0.001) and 10.0 mg/kg cocaine injections (*F*_*5,131*_ = 3.98; *p* = 0.002), with *Fmr1* ^-/Y^ mice significantly less active than WT mice in the first 15 minutes after 1.0 and 3.0 mg/kg (*p* < 0.05) and in the first 30 minutes after 10.0 mg/kg cocaine (*p* < 0.05; Figure **2D**, Figure **S5**). There was a significant interaction between cocaine dose and genotype (*F*_*3,87*_ = 3.44; *p* = 0.02) on locomotor activity after cocaine injection relative to pre-injection baseline activity: locomotor activity increased less in *Fmr1* ^-/Y^ than in WT mice after 3.0 and 10.0 mg/kg cocaine (*p* < 0.05; Figure **2C**).

#### Aripiprazole

**Figure 3:**
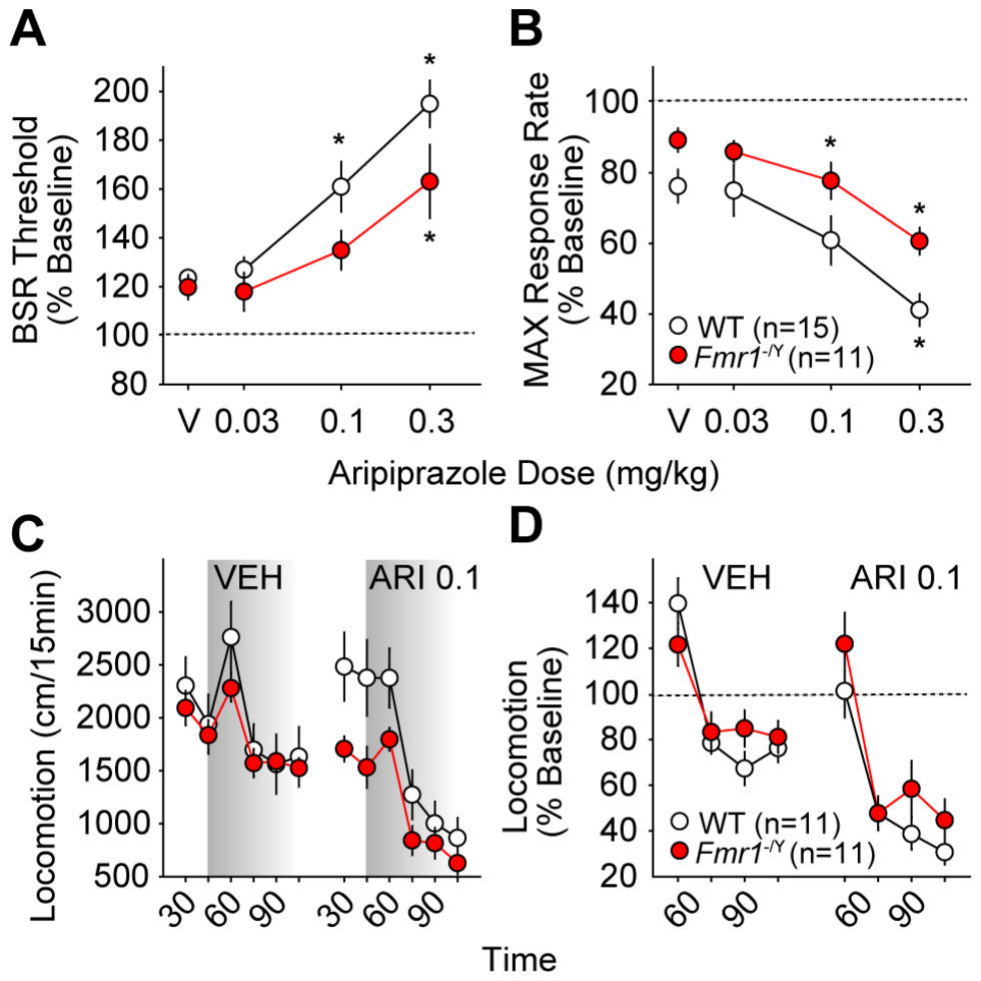
Effects of the atypical neuroleptic aripiprazole on ICSS and locomotor behavior in wild type (WT, *white* *circles*) and *Fmr1* ^-/Y^ mice (red circles). Changes in BSR threshold (**A**) and maximum operant response rate (MAX, **B**) 46-60 minutes after i.p. aripiprazole injections are shown. Values are expressed as mean percentages of pre-injection baselines ± SEM. Asterisks (*) indicate *p* < 0.05 vs. vehicle (V). **C**. Mean distance traveled (± SEM) before and after injection of vehicle (VEH) or aripiprazole (ARI, 0.1 mg/kg i.p.) in 15-minute intervals. Shading indicates post-injection time points. **D**. Mean change in locomotion (± SEM) as a percentage of pre-injection baseline activity for 60 minutes after injection of vehicle (VEH) or aripiprazole (ARI, 0.1 mg/kg i.p.).

Like other neuroleptics, aripiprazole decreases the rewarding value of BSR, evident as elevation of BSR threshold. The onset of aripiprazole effects on ICSS was slow, with maximal effects not seen until after 45-60 minutes following injection (see **Supporting Information**, Figure **S3B**). After 45 minutes post-injection, aripiprazole elevated BSR threshold significantly less in *Fmr1* ^-/Y^ than in WT mice; main effects of dose (*F*_*3,99*_ = 22.09; *p* < 0.001) and genotype (*F*_*1,99*_ = 6.67; *p* = 0.02), but no significant interaction of dose and genotype, were found (Figure **3A**). At this time point the 0.1 and 0.3 mg/kg aripiprazole doses significantly reduced MAX in *Fmr1* ^-/Y^ (*p* < 0.05) but only 0.3 mg/kg reduced MAX in WT mice (*F*_*1,99*_ = 11.02; *p* = 0.003; Figure **3B**, Figure **S4B**).

There was no significant difference between genotypes in total locomotor activity following either vehicle or aripiprazole injection, although *Fmr1* ^-/Y^ mice showed a non-significant trend towards less activity than WT mice after 0.1 mg/kg ARI (*F*_*1,131*_ = 4.21; *p* = 0.053; Figure **3C**, Figures **S6A** and **S6B**). However, relative to pre-injection baseline activity, there were no significant differences in relative reductions of locomotor activity between genotypes following 0.1 mg/kg aripiprazole (Figure **3D**).

#### MPEP

**Figure 4:**
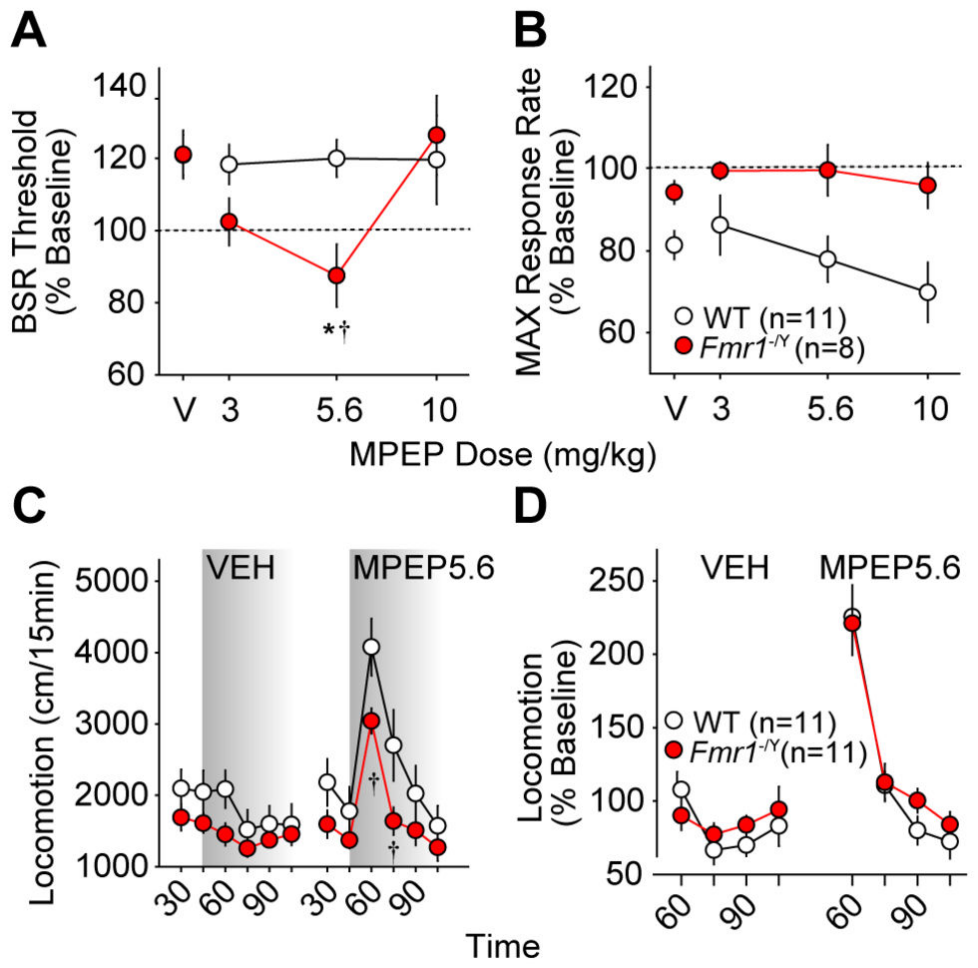
Effects of the mGluR5-selective antagonist MPEP on ICSS and locomotor behavior in wild type (WT, *white* *circles*) and *Fmr1* ^-/Y^ mice (red circles). Changes in BSR threshold (**A**) and maximum operant response rate (MAX, **B**) 31-45 minutes after i.p. MPEP injections are shown. Values are expressed as mean percentages of pre-injection baselines ± SEM. Asterisks (*) indicate *p* < 0.05 vs. vehicle (V); daggers (†) indicate *p* < 0.05 vs. WT (dose x genotype interaction *post* *hoc*). **C**. Mean distance traveled (± SEM) before and after injection of saline (VEH) or MPEP (5.6 mg/kg i.p.) in 15-minute intervals. Shading indicates post-injection time points. Daggers (†) indicate *p* < 0.05 vs. WT (time x genotype interaction *post* *hoc*). **D**. Mean change in locomotion (± SEM) as a percentage of pre-injection baseline for 60 minutes after injection of saline (VEH) or MPEP (5.6 mg/kg i.p.).

Previous studies have shown that MPEP devalues BSR in rats [64], but no prior reports of its effects on ICSS in mice exist. In our hands, MPEP potentiated BSR, and this effect was more evident in *Fmr1* ^-/Y^ than WT mice at 31-45 minutes after MPEP injection (*F*_*3,75*_ = 3.36; *p* = 0.03; Figure **4A**, Figure **S3C**), at which point 5.6 mg/kg MPEP lowered BSR threshold in *Fmr1* ^-/Y^ mice (*p* < 0.05) but no dose of MPEP affected BSR threshold in WT mice. There was a significant main effect of genotype but no effect of MPEP dose on MAX at all time points after injection (Figure **4B**, Figure **S4C** and Table **S2**); regardless of MPEP dose, *Fmr1* ^-/Y^ mice had significantly less reduction in MAX than WT mice.

Total locomotor activity did not differ between genotypes following saline injection, but *Fmr1* ^-/Y^ mice were significantly less active than WT mice (*F*_*5,131*_ = 2.77; *p* = 0.02) in the first 30 min after 5.6 mg/kg MPEP injection (*p* < 0.05; Figure **4C**, Figures **S6C** and **S6D**). However, relative to pre-injection baseline, there was no significant difference in locomotor simulation of *Fmr1* ^-/Y^ and WT mice after 5.6 mg/kg MPEP, with no main effect of genotype and no significant interaction between time and genotype following either saline or MPEP injection (Figure **4D**).

#### Trihexyphenidyl

**Figure 5:**
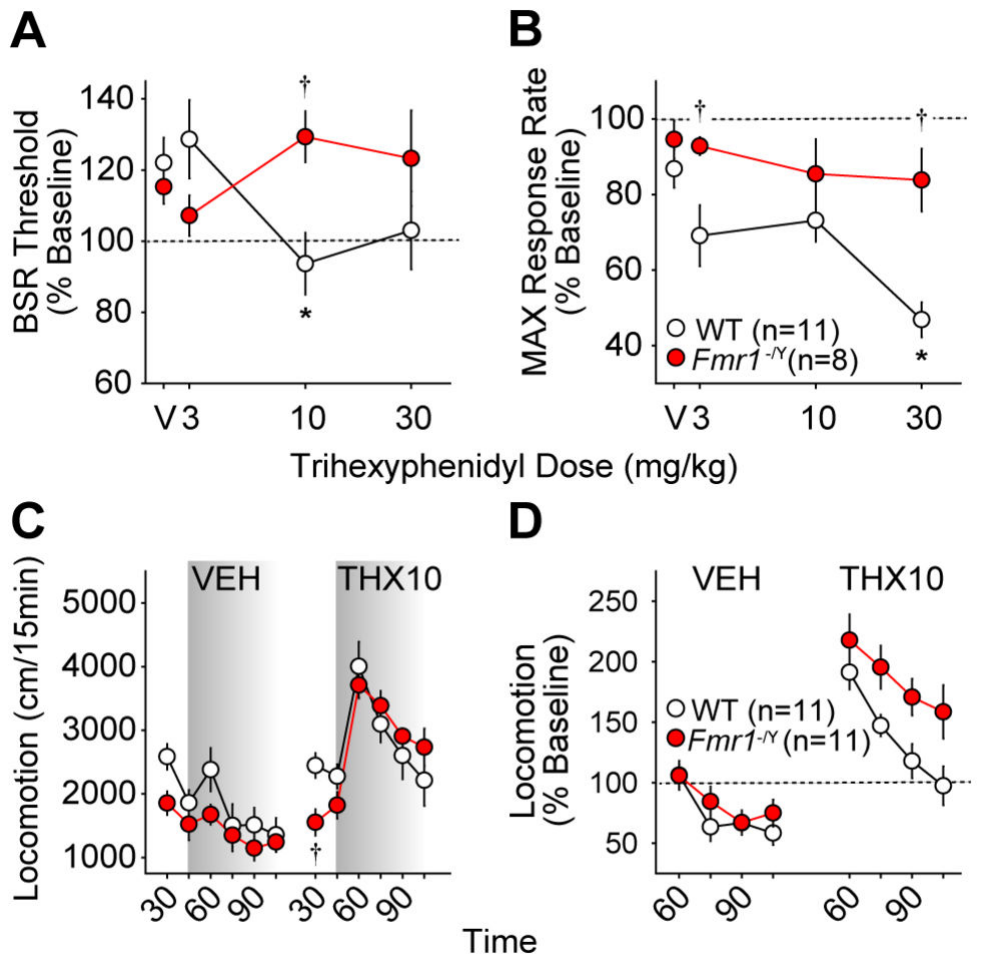
Effects of the partially M1-selective antagonist trihexyphenidyl on ICSS and locomotor behavior in wild type (WT, *white* *circles*) and *Fmr1* ^-/Y^ mice (red circles). Changes in BSR threshold (**A**) and maximum operant response rate (MAX, **B**) 31-45 minutes after i.p. trihexyphenidyl injections are shown. Values are expressed as mean percentages of pre-injection baselines ± SEM. Asterisks (*) indicate *p* < 0.05 vs. vehicle (V); daggers (†) indicate *p* < 0.05 vs. WT (dose x genotype interaction *post* *hoc*). **C**. Mean distance traveled (± SEM) before and after injection of dH_2_O (VEH) or trihexyphenidyl (THX, 10.0 mg/kg i.p.) in 15-minute intervals. Shading indicates post-injection time points. Daggers (†) indicate *p* < 0.05 vs. WT (time x genotype interaction *post* *hoc*). **D**. Mean change in locomotion (± SEM) as a percentage of pre-injection baseline for 60 minutes after injection of dH_2_O (VEH) or trihexyphenidyl (THX, 10.0 mg/kg i.p.).

There is no previous information available on the specific effects of the partially M1-selective muscarinic antagonist, trihexyphenidyl, on BSR. In our hands, 10 mg/kg trihexyphenidyl consistently lowered BSR threshold in WT mice (*p* < 0.05) but not in *Fmr1* ^-/Y^ mice (Figure **5A**) with a significant interaction of dose and genotype (*F*_*3,75*_ = 4.38; *p* = 0.008). No trihexyphenidyl dose affected BSR threshold in the *Fmr1* ^-/Y^ mice at any time point after injection (Figure **S3D** and Table **S1**). There was also a significant interaction between trihexyphenidyl dose and genotype on MAX in the first 45 minutes after injection; 30.0 mg/kg trihexyphenidyl consistently lowered MAX in WT mice (*p* < 0.05), but MAX was unaffected by any dose in *Fmr1* ^-/Y^ mice (Figure **5B**, Figure **S4D** and Table **S2**).

Total locomotor activity did not differ between genotypes following vehicle injection, and while *Fmr1* ^-/Y^ mice were significantly less active than WT mice during minutes 15-30 of the baseline period preceding 10 mg/kg trihexyphenidyl (time x genotype *F*_*5,131*_ = 3.39; *p* = 0.007; *post hoc* p < 0.05), no genotype differences were found following trihexyphenidyl injection (Figure **5C**, Figures **S6E** and **S6F**). Relative to pre-injection baseline activity, there was no significant difference between genotypes after vehicle injection; however, following 10.0 mg/kg trihexyphenidyl, *Fmr1* ^-/Y^ mice were significantly more stimulated than WT mice (*F*_*1,87*_ = 6.10; *p* = 0.02; Figure **5D**).

### Tyrosine Hydroxylase

**Figure 6:**
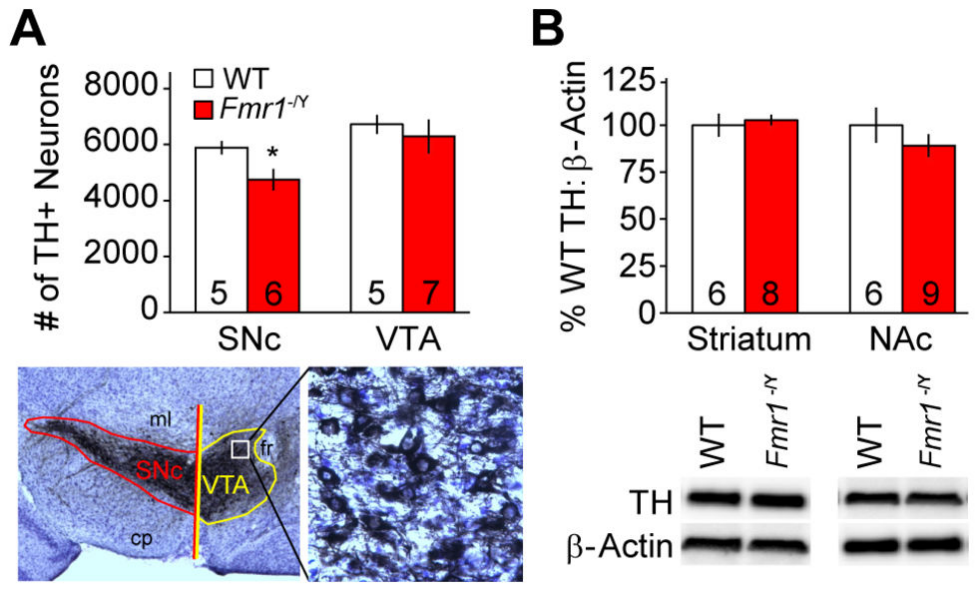
Anatomical and biochemical correlates of dopaminergic function in wild type (WT, *white* *bars*) and *Fmr1* ^-/Y^ mice (red bars). **A**. Mean numbers of tyrosine hydroxylase (TH) expressing neurons (± SEM, *top*) in the substantia nigra pars compacta (SNc) and ventral tegmental area (VTA) of WT and *Fmr1* ^-/Y^ mice estimated with design-based stereology. Numbers of mice are indicated in each column. Ventral midbrain image (bottom left) is magnified 4x and inset showing TH staining (bottom right) 40x. Asterisks (*) indicate *p* < 0.05 vs. WT. cp = cerebral peduncle; fr = fasciculus retroflexus; ml = medial lemniscus. **B**. Mean ratio of TH to β-Actin staining intensity expressed as percentage of mean WT values (± SEM, *top*) in western blots of homogenates from dorsal striatum and nucleus accumbens (NAc) of WT and *Fmr1* ^-/Y^ mice. Numbers of mice are indicated in each column. Representative bands (bottom) are shown for each column above.

Stereological estimates of TH+ neurons in the SNc and VTA of wild-type C57BL6/J mice obtained from our colony were in agreement with previously published values [65]. Fewer TH+ SNc neurons were found in *Fmr1* ^-/Y^ than in WT mice (*t*_*9*_ = 2.56; *p* = 0.03), but numbers of TH+ neurons in the VTA did not differ between genotypes (*t*_*9*_ = -0.12; *p* = 0.91; Figure **6A**). There were no significant differences in TH content of either dorsal striatum (*t*_*12*_ = 0.43; *p* = 0.67) or NAc (*t*_*13*_ = -1.02; *p* = 0.32) between WT and *Fmr1* ^-/Y^ mice (Figure **6B**).

## Discussion

The present study investigated reward and motor function in the *Fmr1* ^-/Y^ mouse model of fragile X syndrome (FXS) by measuring the sensitivity of brain stimulation reward (BSR) and locomotor activity, respectively, to drugs that affect dopamine, muscarinic acetylcholine, and metabotropic glutamate receptors. We also measured the numbers of neurons expressing tyrosine hydroxylase (TH), the rate-limiting enzyme in dopamine biosynthesis, in the midbrain, and measured TH expression in forebrain targets of projections from those neurons. Our data suggest that absence of *Fmr1* does not affect intrinsic sensitivity of mesolimbic circuits to brain stimulation reward (BSR), which we have previously shown to be increased in mice lacking the maternal allele of ubiquitin ligase 3a (*Ube3a* ^*m-/p+*^), a model for Angelman syndrome [55]. However, like *Ube3a* ^*m-/p+*^ mice, *Fmr1* ^-/Y^ mice appear to have reduced activity of nigrostriatal motor circuits that are involved with the initiation of motor activity, expressed as decreased spontaneous locomotion. In contrast to *Ube3a* ^*m-/p+*^ mice, loss of *Fmr1* is associated with reduced numbers of dopaminergic neurons in the substantia nigra pars compacta (SNc). Our behavioral data show that loss of *Fmr1* differentially alters reward and motor responses to specific pharmacological challenges. Drugs used to treat the behavioral symptoms of FXS may differentially impact, and have unintended consequences on, reward activity mediated primarily by mesolimbic circuits and motor activity mediated largely, but not exclusively, through nigrostriatal circuits. Loss of FMRP function may therefore exaggerate some of these differential drug effects.

Although intrinsic sensitivity to BSR was similar between *Fmr1* ^-/Y^ and wild type mice, there were consistent differences in reactions of *Fmr1* ^-/Y^ mice to drug challenges that are known to affect sensitivity to positive reinforcement. In general, *Fmr1* ^-/Y^ mice appeared to be more sensitive to the reward potentiating effects of cocaine and less sensitive to the anhedonic effects of the atypical neuroleptic, aripiprazole. Cocaine potentiation of brain stimulation reward (BSR) is extremely well characterized and robust. While BSR involves distributed neural circuits in the brainstem, basal forebrain, and frontal cortex, dopamine release in the nucleus accumbens (NAc) is necessary for the rewarding effects associated with both BSR and natural incentives such as food, as well as drugs of abuse [66]. Drugs that increase extracellular dopamine availability increase the potency of BSR, measured as a lowering of BSR threshold. Conversely, dopamine antagonists, such as neuroleptics, devalue BSR and raise BSR threshold. While aripiprazole has previously been shown to elevate BSR thresholds in rats [67], this is the first report of its effects on ICSS in a mouse model. The absence of changes in number of ventral tegmental area (VTA) TH+ neurons and TH content in the NAc suggests that structural loss of dopaminergic projections is less likely to account for these findings than functional changes in dopamine release or dopamine receptor function in the NAc and other mesocorticolimbic sites, such as prefrontal cortex (PFC) or the VTA itself.

Dopamine facilitates long-term potentiation (LTP) of excitatory synaptic transmission by increasing synthesis and surface expression of AMPA-type glutamate receptors through D1 receptor signaling [68]. Production of cAMP and enhanced GluA1 phosphorylation following D1 stimulation are reduced in PFC and striatum of *Fmr1* ^-/Y^ mice and can be rescued with FMRP expression [14]. Glutamatergic PFC projections drive firing of GABAergic medium spiny neurons (MSNs), the principal output cells of both NAc and dorsal striatum. D1 receptors contribute to the development of LTP [69] and mGluR5 receptor signaling contributes to synaptic long-term depression (LTD) in NAc MSNs [70]. Decreasing NAc activity is the principal common neural pathway by which reward perception is mediated and translated into motivated behavior [71,72]. It is therefore possible that the enhanced potency of cocaine to potentiate BSR is due to a combination of decreased excitatory drive from PFC to NAc and/or decreased intrinsic excitability of NAc medium spiny neurons in *Fmr1* ^-/Y^ mice. Experiments are currently underway in our laboratory to test these hypotheses.

Normal extrapyramidal motor function requires a balance between dopaminergic and cholinergic activity in the dorsal striatum, such that increasing dopamine or decreasing acetylcholine has hyperkinetic effects and, conversely, decreasing dopamine or increasing acetylcholine has hypokinetic effects. Both the reduced initial locomotion and the reduced potency of cocaine to stimulate locomotor activity we observed in *Fmr1* ^-/Y^ mice is consistent with decreased numbers of dopaminergic neurons found in the SNc, although this was not reflected in decreased TH content in their dorsal striatal targets. However, Fulks et al. [41] have shown that while tissue content of dopamine and its primary metabolites, DOPAC and HVA, is unchanged in the dorsal striatum of *Fmr1* ^-/Y^ mice, electrically-stimulated dopamine release measured with fast-scan cyclic voltammetry in striatal slices is decreased; and this decrease is not associated with changes in D2 autoreceptor function. These findings together with ours predict a parkinsonian motor phenotype in this model, which is observed in older FXS patients. CGG expansion in the FMR1 gene of premutation FXS carriers results in progressive neurodegeneration associated with parkinsonism in fragile X-associated tremor/ataxia syndrome (FXTAS). However, to our knowledge decreased numbers of dopaminergic SNc neurons has not been previously reported either in post-mortem brains of FXS patients or in *Fmr1* ^-/Y^ mice. In summary, our data suggest that dopaminergic function is differentially altered in mesolimbic and nigrostriatal pathways in the *Fmr1* ^-/Y^ mouse model of FXS, the balance of effects facilitating reward function and diminishing motor function. 

While the importance of cholinergic actions in the striatum is appreciated, and anticholinergics have been in routine clinical use for extrapyramidal movement disorders for decades, the function of large, aspiny, tonically active cholinergic interneurons (TANs) remains much less understood than other striatal cell types, such as MSNs and GABAergic fast-spiking interneurons (FSIs), particularly in the NAc. TANs synapse extensively with MSNs and are the primary source of acetylcholine in both the dorsal striatum and NAc. As is the case for their role in motor function, the euphoric and rewarding effects of muscarinic anticholinergics have been appreciated for centuries, but the exact mechanisms by which anticholinergics function in mesolimbic brain reward circuitry are not well understood. Recent studies have suggested that TANs may integrate thalamostriatal and corticostriatal input [51] and local FSI function [54,73] to regulate firing of MSNs [74]. Most of what is known regarding TAN function has been learned from experiments in the dorsal striatum, and with a few exceptions [52,74] these neurons have been largely uninvestigated in the NAc.

The net behavioral effects of the M1 antagonist, trihexyphenidyl, in WT mice were potentiation of BSR within a narrow dose range, suppression of maximum operant response rate without a change in threshold at the highest dose tested (30 mg/kg), and locomotor stimulation at a dose that potentiated BSR (10 mg/kg). In contrast, trihexyphenidyl did not potentiate BSR in *Fmr1* ^-/Y^ mice at any dose, and the locomotor stimulation we observed at 10 mg/kg was significantly enhanced in *Fmr1* ^-/Y^ compared to WT mice, consistent with a parkinsonian motor phenotype and further illustrating the dissociation of reward and motor effects in *Fmr1* ^-/Y^ mice. Trihexyphenidyl has no effect by itself on dopamine release in the NAc, but increases cocaine-potentiated NAc dopamine release and cocaine-stimulated locomotor activation [53], suggesting that cholinergic regulation of mesolimbic circuitry normally keeping NAc dopamine release in check is disinhibited by M1 antagonism, and that this function may be impaired in *Fmr1* ^-/Y^ mice. Acetylcholine signaling through M2/M4 receptors on TANs indirectly reduces dopamine release [52], but signaling through M1 receptors on presynaptic terminals increases excitability of dopaminergic neurons by inhibiting local GABA release in the VTA [75]. Neither mechanism fully explains our present behavioral results with trihexyphenidyl. We are currently investigating how M1 activity in NAc MSNs differs between *Fmr1* ^-/Y^ and WT mice. Given that M1 receptors are also Gq-coupled and signal through PLC, it is possible that M1 function is also altered in NAc MSNs of mice lacking FMRP.

Group I mGluR receptors (mGluR1/5) are distributed ubiquitously throughout the brain and act as a brake on glutamatergic excitation. The highly selective mGluR5 antagonist, MPEP, has anxiolytic potency in rodent models and potentiated BSR in our hands, with larger effects in *Fmr1* ^-/Y^ than in WT mice. MPEP also transiently but significantly stimulated locomotor activity, although relative motor stimulation compared to baseline activity was similar between genotypes. In the dorsal striatum, mGluR5 receptors mediate both postsynaptic AMPA receptor endocytosis [76], which is enhanced in hippocampal neurons of *Fmr1* ^-/Y^ mice [77], and presynaptic endocannabinoid (eCB)-dependent LTD [78]. It has been shown that mGluR5-coupled retrograde eCB signaling through Gq-coupled activation of PLC and diacylglycerol lipase is disrupted in both dorsal striatum [79] and NAc [80], and, in contrast to hippocampus, mGluR5-dependent LTD is absent from NAc MSNs in *Fmr1* ^-/Y^ mice [80], a finding that we have replicated in our laboratory (unpublished observations). In contrast to MSNs, mGluR-dependent LTD is expressed postsynaptically in dopaminergic VTA neurons through increased synthesis and membrane insertion of AMPA receptor GluA2 subunits [81], and preliminary data in our laboratory also suggest that mGluR5-LTD in VTA neurons is enhanced in *Fmr1* ^-/Y^ mice (unpublished observations). Thus, the predicted actions of MPEP on reward and reinforcement are complex, and while mGluR5 activity acts to decrease glutamatergic excitation in different cell types through both pre- and postsynaptic mechanisms, the effect of loss of FMRP on mGluR mechanisms, and ultimately on neuronal activity, likely differs in different elements of brain reward circuitry. Experiments are currently underway in our laboratory investigating both mGluR5 and mAChR1 activity in the VTA and NAc to test these hypotheses.

Although systemic drug administration cannot determine exact mechanisms for the differences we observe in *Fmr1* ^-/Y^ mice, our current findings replicate those of other behavioral and electrophysiological studies reporting that *Fmr1* deletion affects the dopaminergic, glutamatergic, and cholinergic neurotransmitter systems and extend these differences to the regulation of brain reward, which may have clinical implications for patients with FXS. Behavioral therapy is a mainstay in the treatment of children with neurodevelopmental disorders, including FXS. Discrete trial-based learning [82] is a widely-employed therapeutic method in which specific desirable behaviors are rewarded and this approach, by definition, relies upon intact brain mechanisms of reward perception and their ability to reinforce specific behaviors so that the motivation to engage in these behaviors is subsequently enhanced. Many drugs used in the treatment of patients with neurodevelopmental disabilities influence limbic motor function such that their concomitant use could reduce the effectiveness of behavioral therapies by interfering with reward perception or behavioral motivation. Our preclinical findings suggest that the effects of these drugs may differ in individuals with FXS and may therefore inform clinical practice by suggesting behavioral reinforcement and drug regimens specific to FXS patients. In addition, altered reward processing has important implications not only for how individuals with FXS respond to behaviorally-based therapies but also for their socialization, which could be impacted by deficits in social reward or enhanced social aversion.

The current experiments used acute drug dosing to study behavioral pharmacology in *Fmr1* ^-/Y^ mice, but one of the broader aims of these investigations is to determine if selective drugs acting on dopamine, glutamate, or acetylcholine receptors can ameliorate ongoing abnormal behaviors, with the ultimate goal of developing better treatments for individuals with FXS. mGluR5 antagonists are in ongoing clinical trials in FXS patients, but to date no controlled clinical trials have been performed with aripiprazole (Abilify™) or anticholinergics approved for human use, such as trihexyphenidyl (Artane™) or benztropine (Cogentin™). It is unclear if any one drug acting at any one receptor will reduce all FXS symptoms, and more likely that effects at more than one drug target will be necessary to correct different aspects of abnormal behaviors. It is also unclear if drug treatments will need to be continuously administered or if beneficial adaptations to time-limited drug therapy will persist, and at what developmental age or ages such drug therapies will be effective. Preclinical investigation of both basic neural mechanisms and the effects of drugs on behavior in the *Fmr1* ^-/Y^ mouse model remains an important tool in drug discovery for FXS.

## Supporting Information

Figure S1 **ICSS electrode placements.** Confirmation of ICSS electrode tip locations. Brains from mice used for ICSS experiments were fixed by intracardiac perfusion under deep pentobarbital anesthesia, removed, sectioned, and stained with cresyl violet for Nissl to determine electrode placements. The most ventral point of each electrode tract was determined by visual inspection. All ICSS electrodes were implanted on the right: tip positions are plotted on the left for WT mice (grey circles) and on the right for Fmr1^-/Y^ mice (red circles) for clarity.
(TIF)

Figure S2 **Baseline BSR threshold stability over course of ICSS experiments.** Daily baseline BSR thresholds in wild type (WT, *white* *circles*) and *Fmr1* ^-/Y^ mice (*red circles*). Pre-injection baseline BSR thresholds remained stable, and no significant changes were observed in mice of either genotype, over the course of the ICSS experiments. Values are expressed as mean daily baseline BSR threshold ± SEM.
(TIF)

Figure S3 **Changes in BSR threshold following acute drug treatments.** Changes in BSR threshold following acute drug treatment in wild type (WT, *white* *circles*) and *Fmr1* ^-/Y^ mice (*red circles*). **A**. Cocaine lowered BSR threshold in a dose-dependent manner to a greater extent in *Fmr1* ^-/Y^ than WT mice. **B**. Conversely, the atypical neuroleptic aripiprazole elevated BSR threshold in a dose-dependent manner less in *Fmr1* ^-/Y^ than WT mice. **C**. The mGluR5-selective antagonist MPEP lowered BSR threshold to a similar extent in *Fmr1* ^-/Y^ and WT mice immediately after injection (0-15 min), but was more effective longer into the 60-minute test session in *Fmr1* ^-/Y^ than in WT mice (31-45 min). **D**. The partially M1-selective antagonist trihexyphenidyl lowered BSR threshold over a narrow dose range (10 mg/kg) in WT mice, but did not change threshold in *Fmr1* ^-/Y^ mice at any dose. All values are expressed as mean percentage of pre-injection baseline BSR threshold ± SEM. Asterisks (*) indicate *p* < 0.05 vs. vehicle (V); daggers (†) indicate *p* < 0.05 vs. WT (dose x genotype interaction *post* *hoc*). Complete statistics are shown in Table **S1**.
(TIF)

Figure S4 **Changes in maximum operant response rate (MAX) following acute drug treatments.** Changes in maximum operant response rate (MAX) following acute drug treatment in wild type (WT, *white* *circles*) and *Fmr1* ^-/Y^ mice (*red circles*). **A**. Cocaine increased MAX only at the highest dose tested (10 mg/kg) in WT but not *Fmr1* ^-/Y^ mice. **B**. The atypical neuroleptic aripiprazole reduced MAX in a dose-dependent manner to a greater extent in WT mice at earlier time points after injection (15-30 min), but to a similar extent in *Fmr1* ^-/Y^ and WT mice thereafter. Significant main effects of both aripiprazole dose and genotype but no interactions of dose and genotype on MAX were found from 31-75 min after injection. **C**. The mGluR5-selective antagonist MPEP did not significantly affect MAX in either *Fmr1* ^-/Y^ or WT mice. Significant main effects of genotype but no effects of MPEP dose or interactions of dose and genotype on MAX were found at all four post-injection time points. **D**. The partially M1-selective antagonist trihexyphenidyl reduced MAX in a dose-dependent manner in WT but not *Fmr1* ^-/Y^ mice. Significant main effects of genotype on MAX were found at all four post-injection time points after trihexyphenidyl. All values are expressed as mean percentage of pre-injection baseline MAX ± SEM. Asterisks (*) indicate *p* < 0.05 vs. vehicle (V); daggers (†) indicate *p* < 0.05 vs. WT (dose x genotype interaction *post* *hoc*). Complete statistics are shown in Table **S2**.
(TIF)

Figure S5 **Changes in total distance traveled following acute cocaine.** Locomotor behavior before and 60 minutes following acute cocaine administration (COC, 1.0, 3.0, or 10.0 mg/kg i.p.) in wild type (WT, *white* *circles*) and *Fmr1* ^-/Y^ mice (*red circles*). Data are expressed as mean total distance traveled ± SEM in 1 min intervals. Shading indicates post-injection time points.
(TIF)

Figure S6 **Changes in total distance traveled following acute drug treatments.** Locomotor behavior before and 60 minutes following acute drug (*right panels*) or vehicle administration (*left panels*) in wild type (WT, *white* *circles*) and *Fmr1* ^-/Y^ mice (*red circles*). **A**,**B**. Distance traveled after injection of the atypical neuroleptic aripiprazole (ARI, 0.1 mg/kg i.p.) or its vehicle (2% Tween 20). **C**,**D**. Distance traveled after injection of the mGluR5-selective antagonist MPEP (5.6 mg/kg i.p.) or saline (VEH). **E**,**F**. Distance traveled after injection of the partially M1-selective antagonist trihexyphenidyl (THX, 10.0 mg/kg i.p.) or its vehicle (dH_2_O). Data are expressed as mean total distance traveled ± SEM in 1 min intervals. Shading indicates post-injection time points.
(TIF)

Table S1 **Statistical analysis of changes in BSR threshold following acute drug treatments.** Statistical analysis of changes in BSR threshold following acute treatment with cocaine, aripiprazole, MPEP, and trihexyphenidyl, data for which are shown in Figure **S3**. Two-way ANOVA (dose x genotype) with one repeated measure (dose) was performed at each 15 minute testing interval after drug injection. df = degrees of freedom; *n* = number of comparisons.
(DOC)

Table S2 **Statistical analysis of changes in MAX following acute drug treatments.** Statistical analysis of changes in maximum operant response rate (MAX) following acute treatment with cocaine, aripiprazole, MPEP, and trihexyphenidyl, data for which are shown in Figure **S4**. Two-way ANOVA (dose x genotype) with one repeated measure (dose) was performed at each 15 minute testing interval after drug injection. df = degrees of freedom; *n* = number of comparisons.
(DOC)
