# Supplementary figures and images for: Changes in Sensitivity of Reward and Motor Behavior to Dopaminergic, Glutamatergic, and Cholinergic Drugs in a Mouse Model of Fragile X Syndrome
Source: PLoS One. 2013 Oct 18;8(10):e77896. doi: 10.1371/journal.pone.0077896 (PMC3799757; doi:10.1371/journal.pone.0077896)

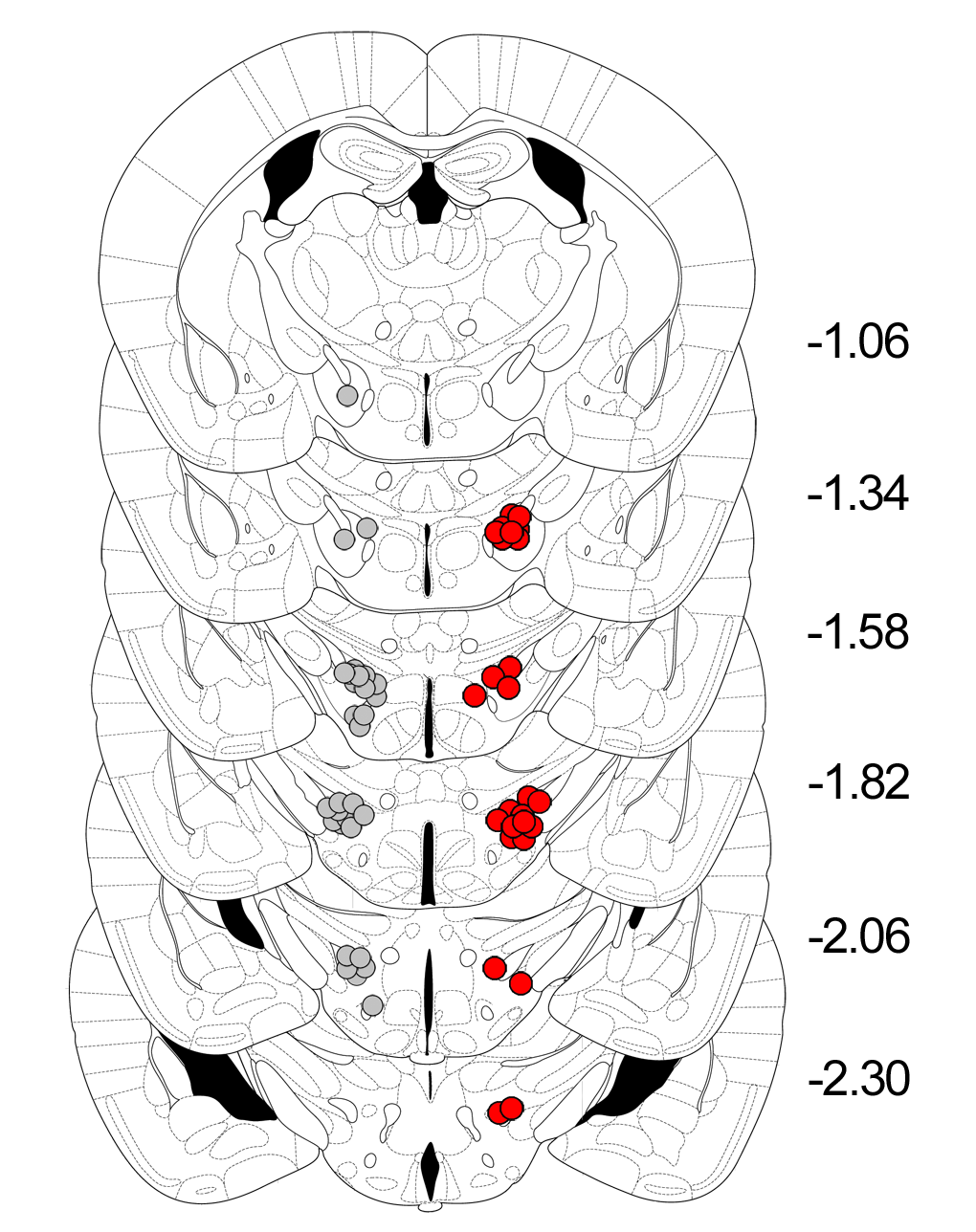

Supplement: Figure S1 — ICSS electrode placements. Confirmation of ICSS electrode tip locations. Brains from mice used for ICSS experiments were fixed by intracardiac perfusion under deep pentobarbital anesthesia, removed, sectioned, and stained with cresyl violet for Nissl to determine electrode placements. The most ventral point of each electrode tract was determined by visual inspection. All ICSS electrodes were implanted on the right: tip positions are plotted on the left for WT mice (grey circles) and on the right for Fmr1-/Y mice (red circles) for clarity. (TIF) [file pone.0077896.s001.tif]

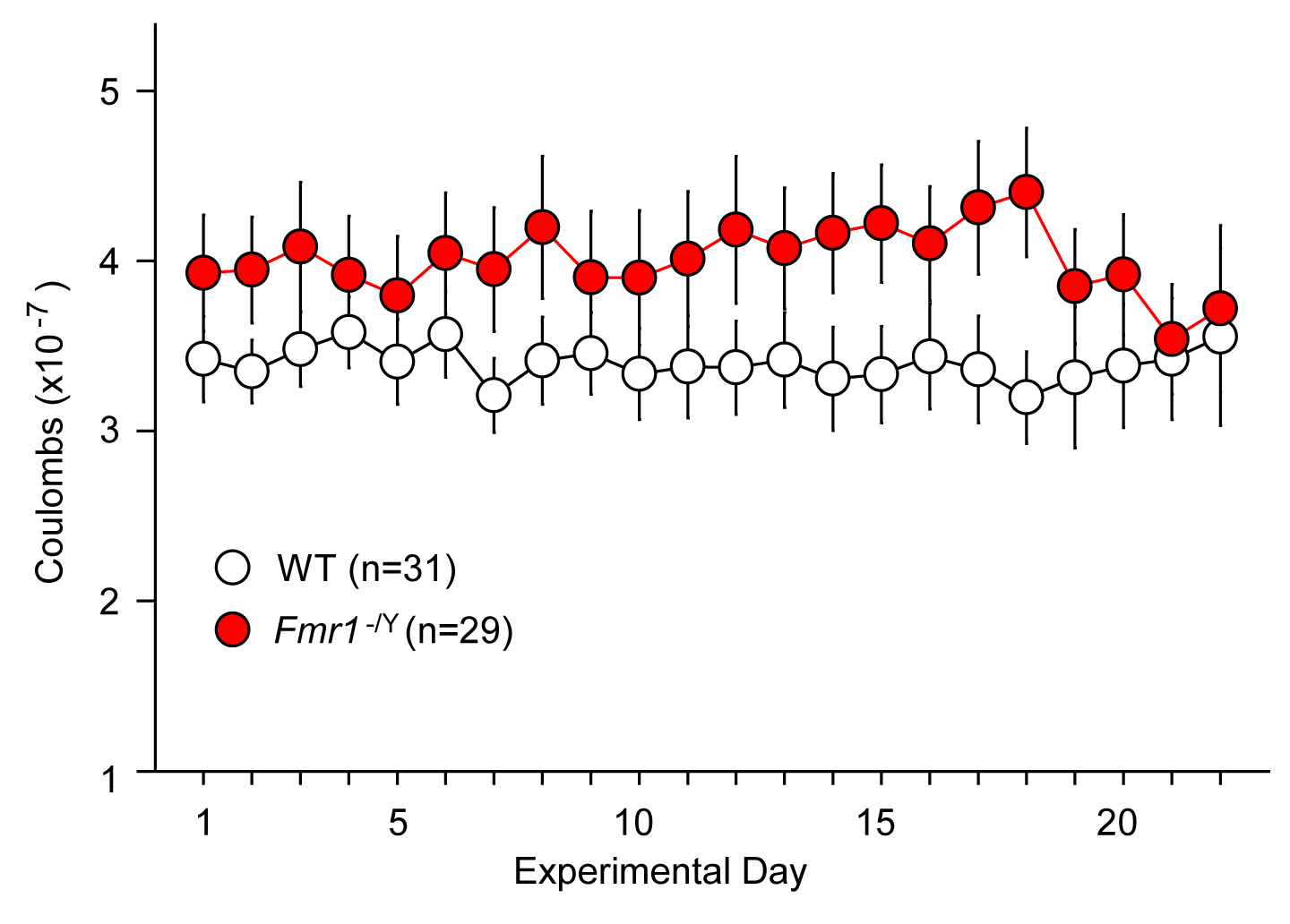

Supplement: Figure S2 — Baseline BSR threshold stability over course of ICSS experiments. Daily baseline BSR thresholds in wild type (WT, white circles) and Fmr1 -/Y mice (red circles). Pre-injection baseline BSR thresholds remained stable, and no significant changes were observed in mice of either genotype, over the course of the ICSS experiments. Values are expressed as mean daily baseline BSR threshold ± SEM. (TIF) [file pone.0077896.s002.tif]

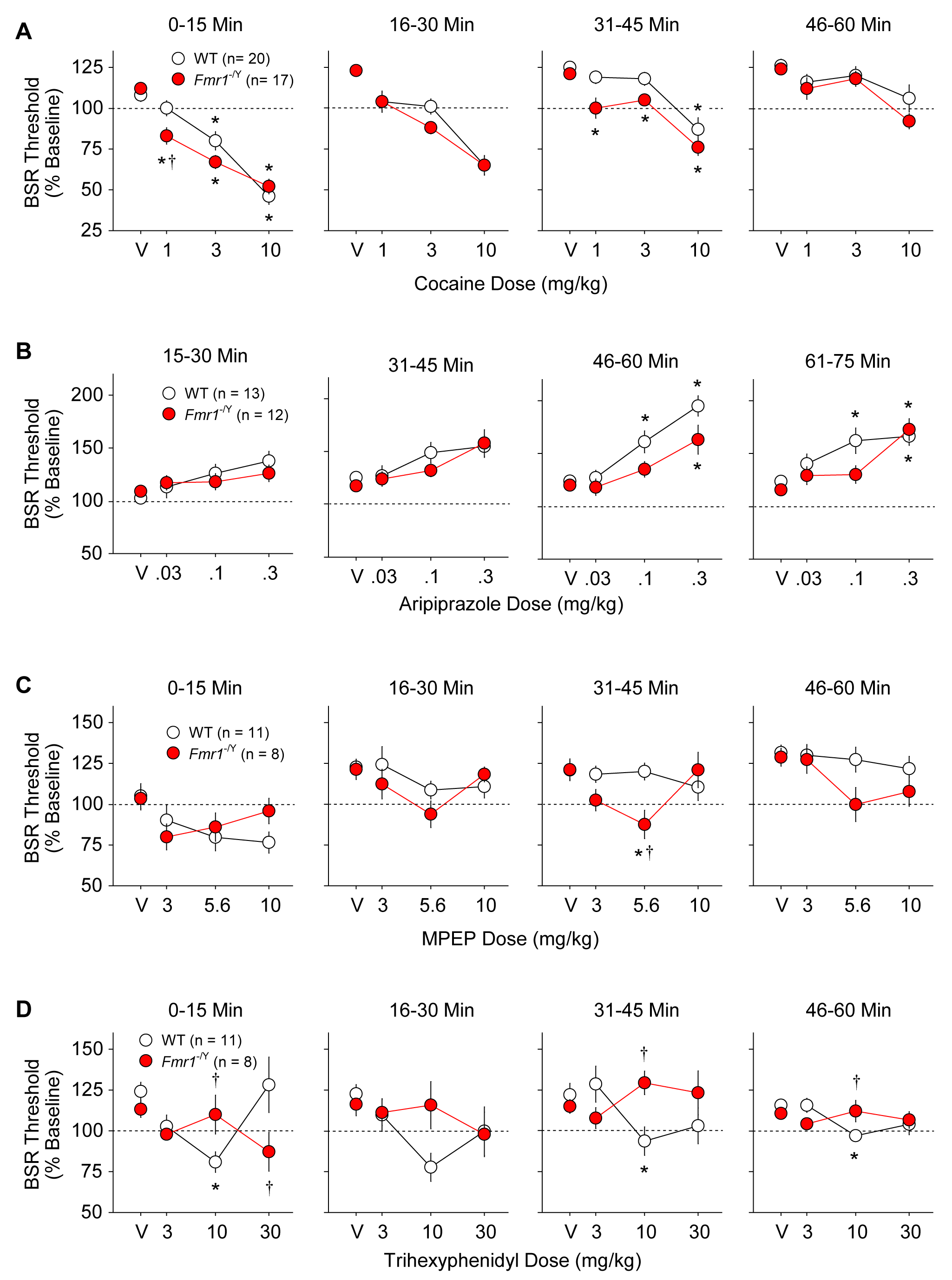

Supplement: Figure S3 — Changes in BSR threshold following acute drug treatments. Changes in BSR threshold following acute drug treatment in wild type (WT, white circles) and Fmr1 -/Y mice (red circles). A. Cocaine lowered BSR threshold in a dose-dependent manner to a greater extent in Fmr1 -/Y than WT mice. B. Conversely, the atypical neuroleptic aripiprazole elevated BSR threshold in a dose-dependent manner less in Fmr1 -/Y than WT mice. C. The mGluR5-selective antagonist MPEP lowered BSR threshold to a similar extent in Fmr1 -/Y and WT mice immediately after injection (0-15 min), but was more effective longer into the 60-minute test session in Fmr1 -/Y than in WT mice (31-45 min). D. The partially M1-selective antagonist trihexyphenidyl lowered BSR threshold over a narrow dose range (10 mg/kg) in WT mice, but did not change threshold in Fmr1 -/Y mice at any dose. All values are expressed as mean percentage of pre-injection baseline BSR threshold ± SEM. Asterisks (*) indicate p < 0.05 vs. vehicle (V); daggers (†) indicate p < 0.05 vs. WT (dose x genotype interaction post hoc). Complete statistics are shown in Table S1 . (TIF) [file pone.0077896.s003.tif]

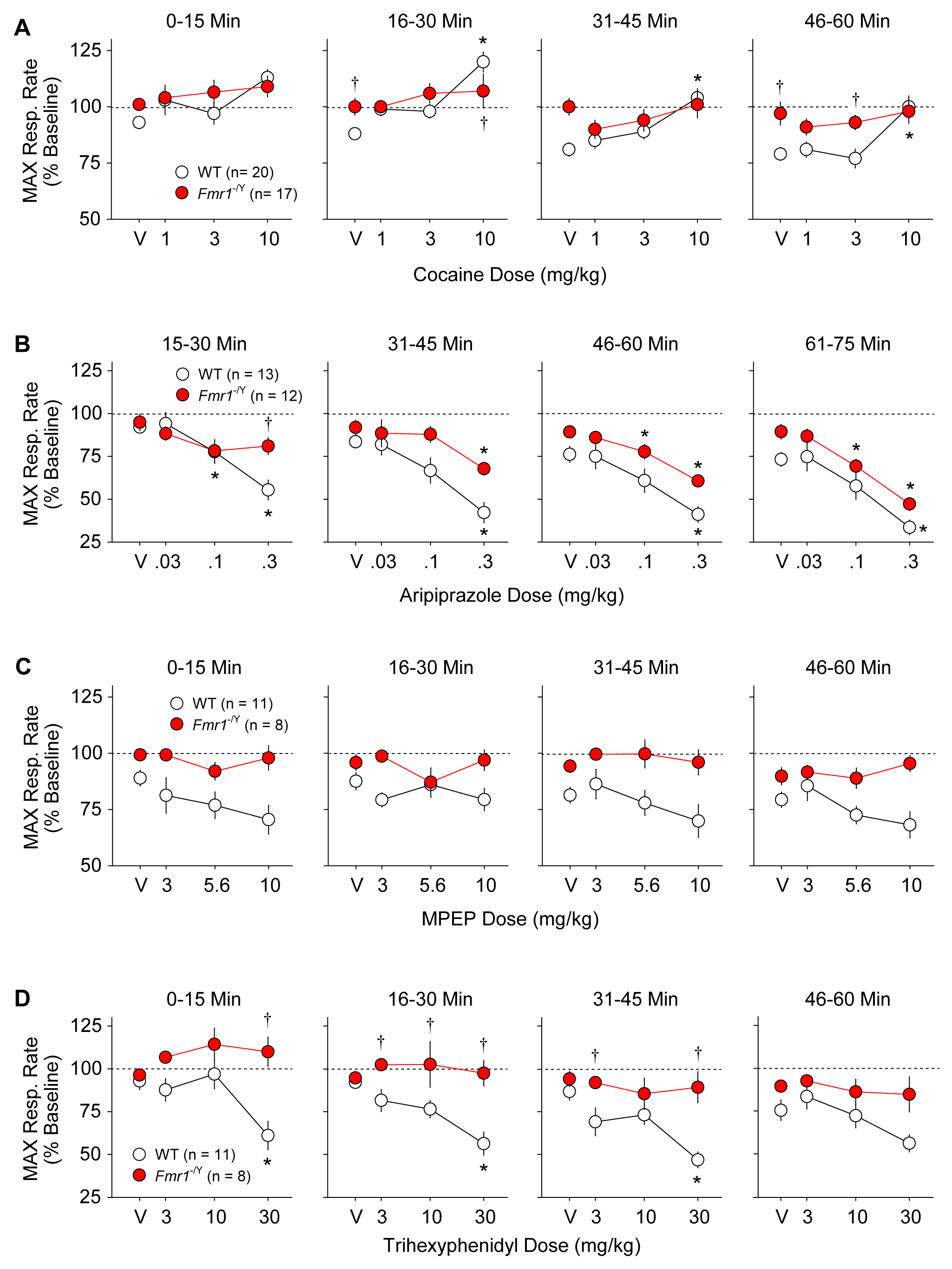

Supplement: Figure S4 — Changes in maximum operant response rate (MAX) following acute drug treatments. Changes in maximum operant response rate (MAX) following acute drug treatment in wild type (WT, white circles) and Fmr1 -/Y mice (red circles). A. Cocaine increased MAX only at the highest dose tested (10 mg/kg) in WT but not Fmr1 -/Y mice. B. The atypical neuroleptic aripiprazole reduced MAX in a dose-dependent manner to a greater extent in WT mice at earlier time points after injection (15-30 min), but to a similar extent in Fmr1 -/Y and WT mice thereafter. Significant main effects of both aripiprazole dose and genotype but no interactions of dose and genotype on MAX were found from 31-75 min after injection. C. The mGluR5-selective antagonist MPEP did not significantly affect MAX in either Fmr1 -/Y or WT mice. Significant main effects of genotype but no effects of MPEP dose or interactions of dose and genotype on MAX were found at all four post-injection time points. D. The partially M1-selective antagonist trihexyphenidyl reduced MAX in a dose-dependent manner in WT but not Fmr1 -/Y mice. Significant main effects of genotype on MAX were found at all four post-injection time points after trihexyphenidyl. All values are expressed as mean percentage of pre-injection baseline MAX ± SEM. Asterisks (*) indicate p < 0.05 vs. vehicle (V); daggers (†) indicate p < 0.05 vs. WT (dose x genotype interaction post hoc). Complete statistics are shown in Table S2 . (TIF) [file pone.0077896.s004.tif]

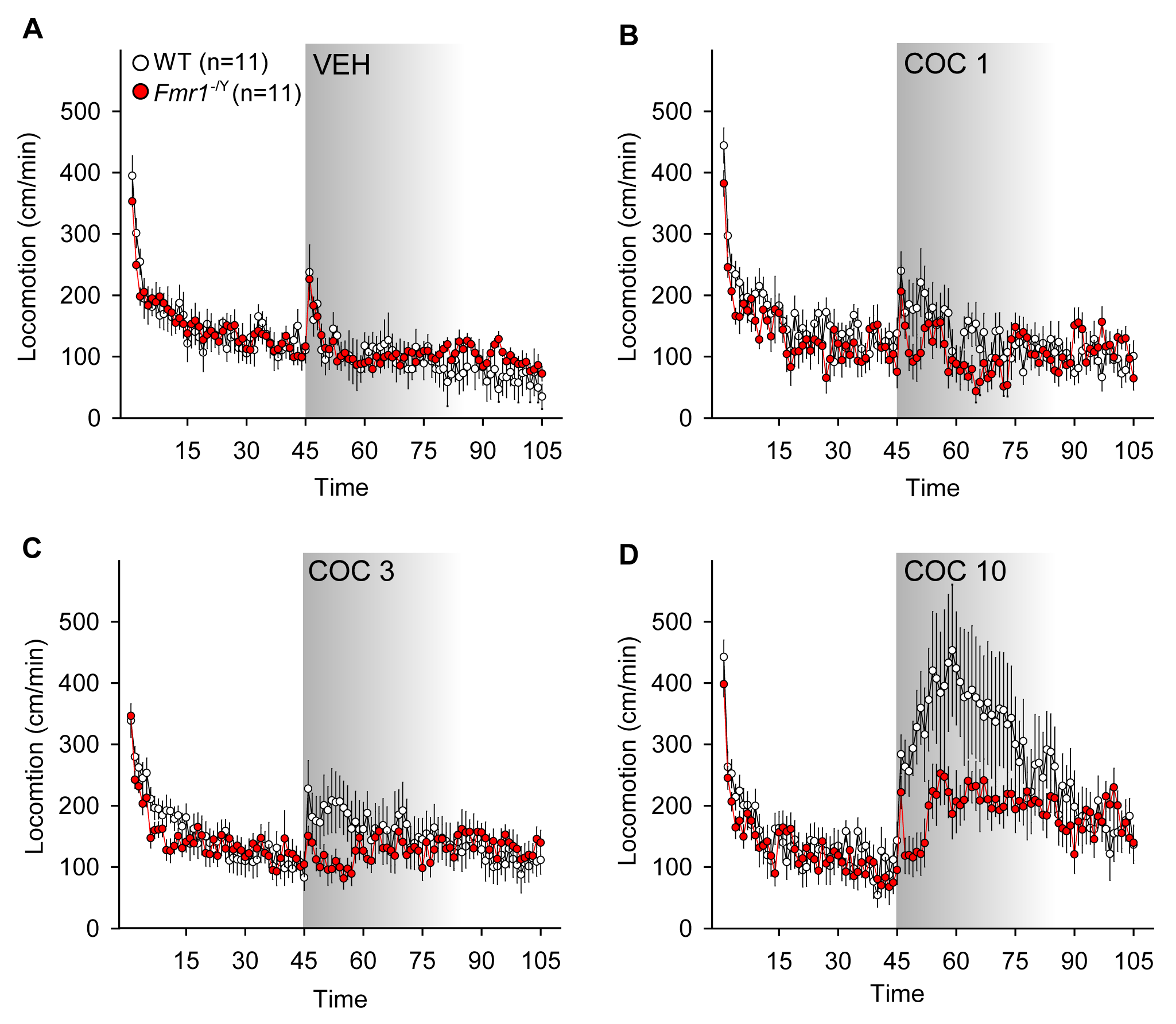

Supplement: Figure S5 — Changes in total distance traveled following acute cocaine. Locomotor behavior before and 60 minutes following acute cocaine administration (COC, 1.0, 3.0, or 10.0 mg/kg i.p.) in wild type (WT, white circles) and Fmr1 -/Y mice (red circles). Data are expressed as mean total distance traveled ± SEM in 1 min intervals. Shading indicates post-injection time points. (TIF) [file pone.0077896.s005.tif]

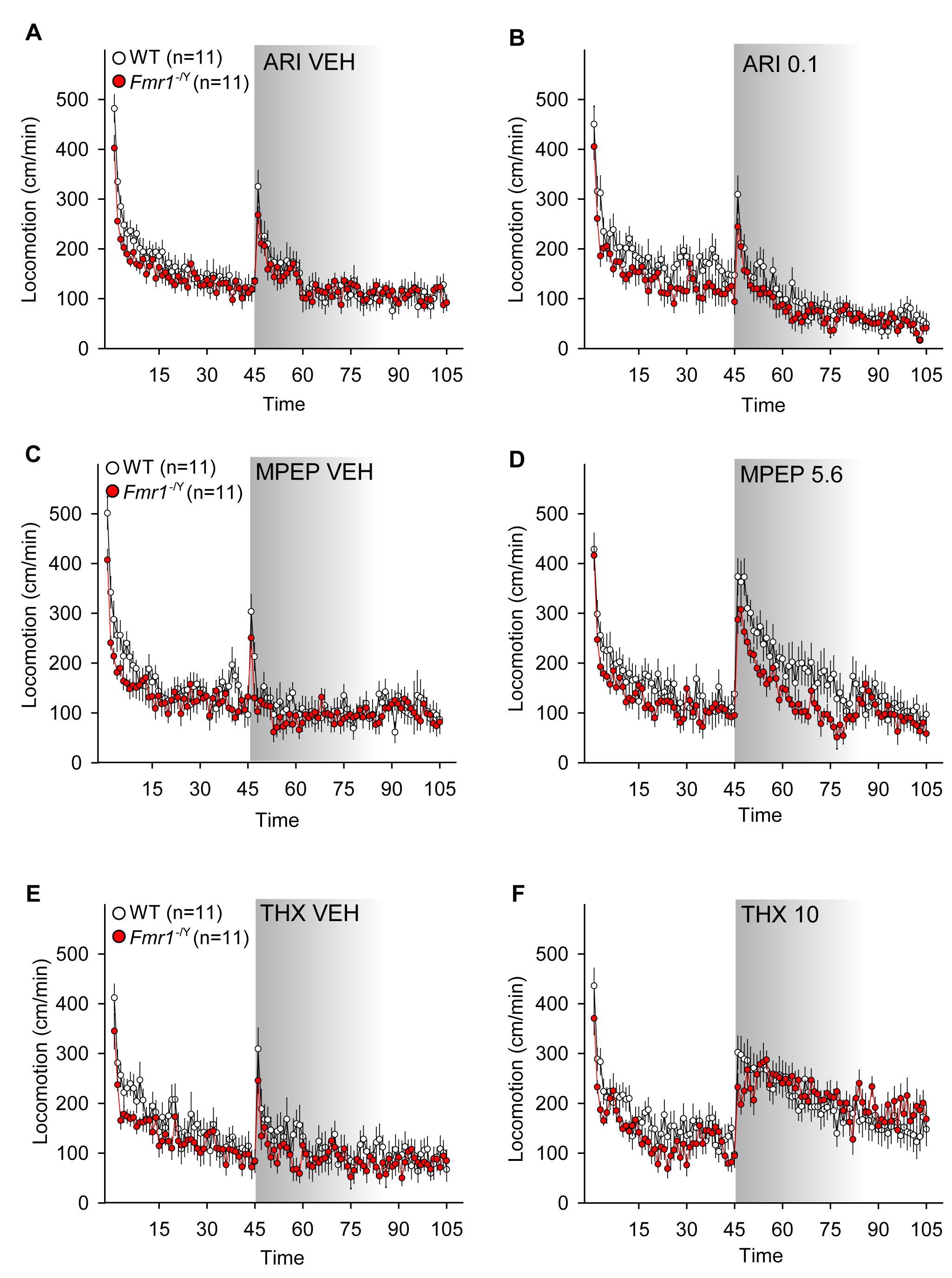

Supplement: Figure S6 — Changes in total distance traveled following acute drug treatments. Locomotor behavior before and 60 minutes following acute drug (right panels) or vehicle administration (left panels) in wild type (WT, white circles) and Fmr1 -/Y mice (red circles). A,B. Distance traveled after injection of the atypical neuroleptic aripiprazole (ARI, 0.1 mg/kg i.p.) or its vehicle (2% Tween 20). C,D. Distance traveled after injection of the mGluR5-selective antagonist MPEP (5.6 mg/kg i.p.) or saline (VEH). E,F. Distance traveled after injection of the partially M1-selective antagonist trihexyphenidyl (THX, 10.0 mg/kg i.p.) or its vehicle (dH2O). Data are expressed as mean total distance traveled ± SEM in 1 min intervals. Shading indicates post-injection time points. (TIF) [file pone.0077896.s006.tif]
