# Supplementary material for: Changes in Sensitivity of Reward and Motor Behavior to Dopaminergic, Glutamatergic, and Cholinergic Drugs in a Mouse Model of Fragile X Syndrome
Source: PLoS One. 2013 Oct 18;8(10):e77896. doi: 10.1371/journal.pone.0077896 (PMC3799757; doi:10.1371/journal.pone.0077896)
Supplement: Table S1 — Statistical analysis of changes in BSR threshold following acute drug treatments. Statistical analysis of changes in BSR threshold following acute treatment with cocaine, aripiprazole, MPEP, and trihexyphenidyl, data for which are shown in Figure S3 . Two-way ANOVA (dose x genotype) with one repeated measure (dose) was performed at each 15 minute testing interval after drug injection. df = degrees of freedom; n = number of comparisons. (DOC) [file pone.0077896.s007.doc]

| Drug | Effect |  | BSR Threshold changes | | | | | | | |
| --- | --- | --- | --- | --- | --- | --- | --- | --- | --- | --- |
|  |  |  | 0-15 minutes | | 16-30 minutes | | 31-45 minutes | | 46-60 minutes | |
|  |  | (*df*, *n*) | *F* | *p* | *F* | *p* | *F* | *p* | *F* | *p* |
| Cocaine | Dose | (3,147) | 70.39^a^ | <*.001*^*^ | 44.78 | *<.001^*^* | 26.36 | *<.001^*^* | 8.69 | *<.001^*^* |
|  | Genotype | (1,147) | 1.99 | .17 | 1.14 | .29 | 13.95 | *<.001^*^* | 1.63 | .21 |
|  | Interaction | (3,147) | 3.29 | *.02*^*^ | 0.96 | .41 | 0.80 | .50 | 0.53 | .66 |
| Aripiprazole | Dose | (3,99) | -- | -- | 4.66 | *.005^*^* | 7.48 | *<.001^*^* | 22.09^b^ | <*.001*^*^ |
|  | Genotype | (1,99) | -- | -- | 0.15 | .70 | 0.98 | .33 | 6.67 | *.02*^*^ |
|  | Interaction | (3,99) | -- | -- | 0.77 | .51 | 0.55 | .65 | 1.37 | .26 |
| MPEP | Dose | (3,75) | 3.89 | *.014^*^* | 2.75 | .052 | 2.06^c^ | .12 | 3.07 | *.036^*^* |
|  | Genotype | (1,75) | 0.22 | .65 | 1.05 | .32 | 4.00 | .06 | 3.73 | .07 |
|  | Interaction | (3,75) | 1.59 | .20 | 0.88 | .46 | 3.36 | *.03*^*^ | 1.35 | .27 |
| Trihexyphenidyl | Dose | (3,75) | 2.27 | .09 | 2.41 | .08 | 0.34^d^ | .80 | 1.45 | .24 |
|  | Genotype | (1,75) | 0.76 | .40 | 0.62 | .44 | 0.72 | .41 | 0.02 | .90 |
|  | Interaction | (3,75) | 4.58 | *.007^*^* | 2.24 | .10 | 4.38 | *.008*^*^ | 2.85 | *.046^*^* |
| ^a^ Data in Figure 2A, ^*^*p* < *0.05*, ^b^ Data in Figure 3A, ^c^ Data in Figure 4A, ^d^ Data in Figure 5A | | | | | | | | | | |
