# Supplementary material for: Changes in Sensitivity of Reward and Motor Behavior to Dopaminergic, Glutamatergic, and Cholinergic Drugs in a Mouse Model of Fragile X Syndrome
Source: PLoS One. 2013 Oct 18;8(10):e77896. doi: 10.1371/journal.pone.0077896 (PMC3799757; doi:10.1371/journal.pone.0077896)
Supplement: Table S2 — Statistical analysis of changes in MAX following acute drug treatments. Statistical analysis of changes in maximum operant response rate (MAX) following acute treatment with cocaine, aripiprazole, MPEP, and trihexyphenidyl, data for which are shown in Figure S4 . Two-way ANOVA (dose x genotype) with one repeated measure (dose) was performed at each 15 minute testing interval after drug injection. df = degrees of freedom; n = number of comparisons. (DOC) [file pone.0077896.s008.doc]

| Drug | Effect |  | MAX operant response rate changes | | | | | | | |
| --- | --- | --- | --- | --- | --- | --- | --- | --- | --- | --- |
|  |  |  | 0-15 minutes | | 16-30 minutes | | 31-45 minutes | | 46-60 minutes | |
|  |  | (*df*, *n*) | *F* | *p* | *F* | *p* | *F* | *p* | *F* | *p* |
| Cocaine | Dose | (3,147) | 3.76^a^ | *0.013*^*^ | 7.97 | *<.001^*^* | 4.74 | *.004^*^* | 5.01 | *.003^*^* |
|  | Genotype | (1,147) | 0.80 | 0.38 | 0.60 | .44 | 6.56 | *.015^*^* | 11.44 | *.002^*^* |
|  | Interaction | (3,147) | 1.07 | 0.37 | 3.43 | *.02^*^* | 2.45 | .07 | 2.74 | *.047^*^* |
| Aripiprazole | Dose | (3,99) | -- | -- | 13.79 | *<.001^*^* | 19.20 | *<.001^*^* | 23.33^b^ | <*.001*^*^ |
|  | Genotype | (1,99) | -- | -- | 1.15 | .30 | 8.00 | *.01^*^* | 11.02 | *.003*^*^ |
|  | Interaction | (3,99) | -- | -- | 4.60 | *.005^*^* | 1.92 | .13 | 0.40 | .75 |
| MPEP | Dose | (3,75) | 2.46 | .07 | 0.60 | .62 | 1.31^c^ | .28 | 1.22 | .31 |
|  | Genotype | (1,75) | 9.19 | *.008^*^* | 6.90 | *.018^*^* | 13.89 | *.002*^*^ | 13.65 | *.002^*^* |
|  | Interaction | (3,75) | 1.39 | .26 | 2.38 | .08 | 0.83 | .49 | 2.11 | .11 |
| Trihexyphenidyl | Dose | (3,75) | 3.45 | *.023^*^* | 3.49 | *.022^*^* | 4.71^d^ | *.006*^*^ | 2.52 | .07 |
|  | Genotype | (1,75) | 10.56 | *.005^*^* | 14.33 | *.001^*^* | 14.82 | *.001*^*^ | 10.64 | *.005^*^* |
|  | Interaction | (3,75) | 4.80 | *.005^*^* | 3.86 | *.015^*^* | 3.36 | *.03*^*^ | 0.82 | .49 |
| ^a^ Data in Figure 2B, ^*^*p* < *0.05*, ^b^ Data in Figure 3B, ^c^ Data in Figure 4B, ^d^ Data in Figure 5B | | | | | | | | | | |
